# Parton distributions with small-*x* resummation: evidence for BFKL dynamics in HERA data

**DOI:** 10.1140/epjc/s10052-018-5774-4

**Published:** 2018-04-20

**Authors:** Richard D. Ball, Valerio Bertone, Marco Bonvini, Simone Marzani, Juan Rojo, Luca Rottoli

**Affiliations:** 1 The Higgs Centre for Theoretical Physics, University of Edinburgh, JCMB, KB, Mayfield Rd, Edinburgh, EH9 3JZ Scotland UK; 2 Department of Physics and Astronomy, VU University, 1081 HV Amsterdam, The Netherlands; 3 Nikhef Theory Group, Science Park 105, 1098 XG Amsterdam, The Netherlands; 4 Dipartimento di Fisica, Sapienza Università di Roma and INFN, Sezione di Roma, Piazzale Aldo Moro 5, 00185 Rome, Italy; 5 Dipartimento di Fisica, Università di Genova and INFN, Sezione di Genova, Via Dodecaneso 33, 16146 Genoa, Italy; 6 Rudolf Peierls Centre for Theoretical Physics, University of Oxford, 1 Keble Road, Oxford, OX1 3NP UK

## Abstract

We present a determination of the parton distribution functions of the proton in which NLO and NNLO fixed-order calculations are supplemented by NLL*x* small-*x* resummation. Deep-inelastic structure functions are computed consistently at $\hbox {NLO+NLL}x$ or $\hbox {NNLO+NLL}x$, while for hadronic processes small-*x* resummation is included only in the PDF evolution, with kinematic cuts introduced to ensure the fitted data lie in a region where the fixed-order calculation of the hard cross-sections is reliable. In all other respects, the fits use the same methodology and are based on the same global dataset as the recent NNPDF3.1 analysis. We demonstrate that the inclusion of small-*x* resummation leads to a quantitative improvement in the perturbative description of the HERA inclusive and charm-production reduced cross-sections in the small *x* region. The impact of the resummation in our fits is greater at NNLO than at NLO, because fixed-order calculations have a perturbative instability at small *x* due to large logarithms that can be cured by resummation. We explore the phenomenological implications of PDF sets with small-*x* resummation for the longitudinal structure function $F_L$ at HERA, for parton luminosities and LHC benchmark cross-sections, for ultra-high-energy neutrino–nucleus cross-sections, and for future high-energy lepton–proton colliders such as the LHeC.

## Introduction

The experiments at CERN’s large hadron collider (LHC) continue to explore particle physics both at the high-energy and the high-precision frontiers. The outstanding quality of current and forthcoming LHC data challenges the theory community to perform more precise calculations, so that meaningful conclusions can be drawn when comparing these theoretical predictions to experimental measurements. In this respect, the tremendous effort to be made in order to arrive at precision calculations for hard-scattering matrix elements and final-state parton evolution has to be accompanied by a comparable level of understanding of the internal structure of the initial-state hadrons.

Global analyses of PDFs [1–6] (see [7–11] for recent overviews) are generally based on fixed-order perturbative calculations, at LO, NLO and NNLO. However, it is well known that further logarithmic enhancements can affect partonic cross-sections and DGLAP evolution kernels order by order in perturbation theory. If we denote by *Q* the hard scale of the process of interest and by $\sqrt{s}$ the center-of-mass energy of the colliding protons, we have logarithmic enhancements in two opposite limits, namely $Q^2 \sim s$ (the threshold region) and $Q^2 \ll s$ (the high-energy region). Introducing the variable $x=Q^2/s$, the threshold limit corresponds to large *x*, while the high-energy limit to small *x*.

The LHC is exploring a vast kinematic range in *x*, potentially covering both extreme regions. It is therefore crucially important to consistently assess the role of logarithmic corrections both at large and small *x*. For instance, searches for new resonances at high mass are sensitive to PDFs in the region between $0.1 \lesssim x \lesssim 0.7$ [12]. On the other hand, processes such as forward production of Drell–Yan lepton pairs at small di-lepton invariant masses [13] and of *D* mesons at small $p_T^D$ [14], both measured by the LHCb collaboration, probe values of *x* at the other end of the spectrum, down to $x\sim 10^{-6}$.

Calculations that aim to describe these extreme regions of phase space should in principle include resummation in the calculations of matrix elements and should make use of PDFs that were determined with a consistent theory. Threshold (large-*x*) resummation has already been included in PDF fits [15] (see also Ref. [16]) and dedicated studies which include threshold resummation in both the coefficient functions and in the PDFs have been performed in the context of heavy supersymmetric particle production [12]. The inclusion of threshold resummation in PDF fits is straightforward because in the widely used $\overline{\text {MS}}$ scheme the DGLAP evolution kernels are not enhanced at large *x* [17, 18], so threshold resummation is only necessary for the coefficient functions, and can thus be included rather easily.

The situation is rather more intricate for small-*x* resummation, because both coefficient functions and splitting functions receive single-logarithmic contributions to all orders in perturbation theory. Small-*x* resummation is based on the BFKL equation [19–23]. However, the naive application of the fixed coupling leading-log *x* (LL*x*) BFKL equation to small-*x* deep-inelastic scattering (DIS) structure functions predicted a much steeper growth than that actually observed by the first HERA measurements [24, 25], which instead were well reproduced by the predictions of LO and NLO running coupling DGLAP [26–31]. This paradox was compounded by the computation of next-to-leading logarithmic (NLL*x*) corrections to the evolution kernels [32–36], which turned out to be large and negative, destabilizing the LL*x* BFKL result. The correct implementation of small-*x* resummation turns out to require the simultaneous resummation of collinear and anti-collinear singularities in the small-*x* evolution kernels, together with a consistent resummation of running coupling effects.

This problem was tackled by several groups; see Refs. [37–46] (ABF), Refs. [47–57] (CCSS) and Refs. [58–61] (TW), which explored various theoretical and phenomenological aspects of the problem, with the goal of achieving consistent and phenomenologically viable frameworks that resum collinear and high-energy logarithms simultaneously. Resummation corrections to fixed-order evolution, when consistently implemented, were shown to be reasonably small, thus explaining the success of the conventional unresummed description used in standard PDF determinations. More recently, small-*x* resummation based on the ABF formalism has been consistently matched to fixed NNLO for perturbative evolution and deep-inelastic structure functions, and implemented in the public code HELL [62, 63], making small-*x* resummation available for phenomenological applications.

On the other hand, while fixed-order DGLAP theory can provide a reasonable fit to the inclusive HERA data, several groups have found indications that the description of the most precise legacy datasets is not optimal in the small-*x* and small-$Q^2$ region, especially at NNLO^1^ [64–70]. Currently, the evidence that this tension is related to lack of small-*x* resummation is inconclusive. The only way to show that it is due to resummation would be to perform a complete global PDF analysis including small-*x* resummation. Since the effect of resummation is known to be small, at least in the kinematic region explored at HERA, it is necessary that these fits are free of methodological bias. The NNPDF framework [71–79], having been validated by a closure test, is thus ideal in this respect.

With these motivations, the goal of this paper is to present a state-of-the-art PDF determination in which NLO and NNLO fixed-order perturbation theory is matched to NLL*x* small-*x* resummation. This will be done by supplementing the recent NNPDF3.1 PDF determination [79] with small-*x* resummation of DGLAP evolution and DIS coefficient functions using HELL, thereby leading to resummed PDF sets. We will show that the inclusion of small-*x* resummation significantly improves the quantitative description of the small-*x* and small-$Q^2$ HERA data, in particular at NNLO, both for the inclusive and for the charm structure functions. Our results fulfill a program that was initiated more than 20 years ago, when the first measurements of $F_2(x,Q^2)$ at HERA stimulated studies on the inclusion of small-*x* resummation in perturbative evolution [80–83].

The outline of this paper is as follows. First, in Sect. 2 we review the implementation of small-*x* resummation that we will use, and illustrate how resummation affects PDF evolution and DIS structure functions. Then in Sect. 3 we present the settings of our fits, which we dub NNPDF3.1sx, and in particular we discuss the choice of kinematic cuts. The results of the fits with small-*x* resummation are discussed in Sect. 4. In Sect. 5 we show the comparisons with the HERA experimental data, and provide detailed evidence for the onset of resummation effects in the inclusive and charm-production structure functions. We then perform a first exploration of the phenomenological implications of the NNPDF3.1sx fits at the LHC and beyond in Sect. 6, and finally in Sect. 7 we summarize and outline possible future developments.

## Implementation of small-*x* resummation

Here we briefly review the implementation of small-*x* resummation which will be adopted in the sequel. First, we summarize the general features of small-*x* resummation theory, its main ingredients, and available approaches to it. We then discuss separately the implementation and general phenomenology of small-*x* resummation of perturbative evolution, and of deep-inelastic structure functions.

### Basics of small-*x* resummation

In collinear factorization, the deep-inelastic scattering structure functions can be expressed as

2.1 $$\begin{aligned} \sigma (x,Q^2)= & {} x \sum _{i}\int _x^1 {{\mathrm{d}z}\over {z}}\, \sigma _0\left( Q^2,\alpha _s\left( \mu _\text {R}^2\right) \right) \nonumber \\&\times C_{i}\left( z,\alpha _s\left( \mu _\text {R}^2\right) ,{{Q^2}\over {\mu _\text {F}^2}},{{Q^2}\over {\mu _\text {R}^2}}\right) \,f_{i}\left( {{x}\over {z}},\mu _\text {F}^2\right) ,\nonumber \\ \end{aligned}$$

where $x=Q^2/s$, $\mu _\text {R}$ and $\mu _\text {F}$ are the renormalization and factorization scales, the sum runs over partons, and we have factored out for convenience the Born cross-section $\sigma _0$. Similarly for hadronic processes

2.2 $$\begin{aligned} \sigma (x,M^2)= & {} x \sum _{ij}\int _x^1 {{\mathrm{d}z}\over {z}}\, \sigma _0\left( M^2,\alpha _s\left( \mu _\text {R}^2\right) \right) \nonumber \\&\times C_{ij}\left( z,\alpha _s\left( \mu _\text {R}^2\right) ,{{M^2}\over {\mu _\text {F}^2}},{{M^2}\over {\mu _\text {R}^2}}\right) \,\mathcal{L}_{ij}\left( {{x}\over {z}},\mu _\text {F}^2\right) ,\nonumber \\ \end{aligned}$$

where $M^2$ is the invariant mass of the particles produced in the final state, $x=M^2/s$, and the parton luminosities

2.3 $$\begin{aligned} \mathcal {L}_{ij}(z,\mu ^2) = \int _z^1 {{\mathrm{d} w}\over {w}} \, f_i\left( {{z}\over {w}},\mu ^2\right) f_j(w,\mu ^2). \end{aligned}$$

The scale dependence of the PDFs $f_{i}\left( x,\mu ^2\right)$ is controlled by the DGLAP evolution equations

2.4 $$\begin{aligned} \mu ^2 {{\partial }\over {\partial \mu ^2}} f_i(x,\mu ^2) = \int _x^1 {{\mathrm{d} z}\over {z}} P_{ij}\left( {{x}\over {z}},\alpha _s(\mu ^2) \right) f_j(z,\mu ^2), \end{aligned}$$

and knowledge of the splitting kernels $P_{ij}(x,\alpha _s)$ to $(k+1)$-loops allows for the resummation of collinear logarithms at $\hbox {N}^k\hbox {LO}$ accuracy. The evolution kernels are currently known to NNLO (3 loops) [84, 85], and partially even to $\hbox {N}^3\hbox {LO}$ (4 loops) [86, 87].

Single logarithms of *x* affect higher order corrections to both splitting functions and hard cross-sections. Specifically, the generic all-order behavior of the gluon–gluon splitting function is $P_{gg}\sim {{1}\over {x}}\sum _n \alpha _s^{n} \ln ^{n-1}{{1}\over {x}}$. Small-*x* logarithms are mostly relevant for PDFs in the singlet sector, i.e. the gluon and the quark singlet: small-*x* (double) logarithms in nonsinglet PDFs are suppressed by an extra power of *x*. Partonic cross-sections (either inclusive, or differential in rapidity or transverse momentum) can also contain small-*x* logarithms, which depend on the process and the observable. For gluon-induced processes (such as Higgs or top production) resummation affects the leading-order cross-section and it is thus a leading-log *x* (LL*x*) effect, while for quark-induced processes (such as Drell–Yan or deep-inelastic scattering) there must be a gluon-to-quark conversion, which makes it a NLL*x* effect. In either event at small *x* and low scales the combination $\alpha _s\ln {{1}\over {x}}$ can become large, spoiling fixed-order perturbation theory. In these circumstances it becomes necessary to resum the large logarithms in both splitting and coefficient functions in order to obtain reliable predictions.

Small-*x* resummation is based on the BFKL equation [19–23], which can be written as an evolution equation in *x* for off-shell gluons. Knowledge of the BFKL kernel *K* to $(k+1)$-loops allows for the resummation of small-*x* logarithms to $\hbox {N}^k\hbox {LL}x$. The BFKL kernel is currently known to 2 loops [32–36], and to 3 loops in the collinear approximation [88] (see Refs. [89–94] for other recent works on extending BFKL beyond NLL*x*). Thus, with current technology small-*x* logarithms can be fully resummed to NLL*x* accuracy.

A simultaneous resummation of collinear and high-energy logarithms can be obtained if one consistently combines the DGLAP and BFKL equations. However, it turns out that this is far from trivial, particularly when the coupling runs, since the BFKL kernel also contains collinear (and anti-collinear) singularities which must be matched to those in DGLAP. This problem received great attention from several groups: Altarelli, Ball and Forte [37–46], Ciafaloni, Colferai, Salam and Stasto [47–57] and Thorne and White [58–61], each of which produced resummed splitting functions for PDF evolution. In the end, the theoretical ingredients used by the various groups were similar, thus leading to compatible results (for a detailed comparison between the different approaches see [95, 96]). More recently, a public code named HELL (High-Energy Large Logarithms) [62, 63] has been produced to perform small-*x* resummation to NLL*x* of singlet splitting functions matched to NLO and NNLO fixed-order evolution. HELL is largely based on the formalism developed by Altarelli, Ball and Forte (ABF) [37–46].

In the ABF approach, one constructs perturbatively stable resummed results by combining three main ingredients: duality, i.e. consistency relations between the DGLAP and BFKL evolution kernels [37, 38, 97, 98], which are used to construct a double-leading evolution kernel that simultaneously resums both collinear and small-*x* logarithms; symmetrization of the BFKL kernel in order to stabilize its perturbative expansion both in the collinear and anti-collinear regions of phase space [43, 47], and thus in the region of asymptotically small *x*; and resummation of running coupling contributions, which despite being formally subleading are in fact dominant asymptotically, since they change the nature of the small-*x* singularity [41, 42, 52, 53, 58, 99]. The resummation of gluon evolution with all the above ingredients consistently combined was originally achieved to $\hbox {NLO+NLL}x$ in Refs. [43, 53], while the inclusion of the quark contributions and the rotation to the physical basis of the singlet sector was completed in Refs. [46, 57]. The matching to NNLO has been recently achieved in [63] and represents an important new development since it makes it possible to compare NNLO results with and without NLL*x* small-*x* resummation included.

Thanks to high-energy factorization [100–103] (generalized in Ref. [104] to rapidity and in Refs. [105, 106] to transverse momentum distributions) it is possible to also perform resummation of the leading small-*x* logarithms in the coefficient functions both in deep-inelastic cross-sections Eq. (2.1) and hadronic cross-sections Eq. (2.2). The resummation relies on the resummation of the splitting function, which must then be combined with a computation of the hard cross-section with incoming off-shell gluons. Such calculations have been made for a range of processes: heavy-quark production [100, 101, 107, 108], DIS structure functions [103, 109, 110], Drell–Yan production [111, 112], direct photon production [113, 114] and Higgs production [115–117]. The use of these expressions to resum coefficient functions at fixed coupling is straightforward, but becomes more complicated when the coupling runs, due to the presence of anti-collinear singularities. This issue was resolved (both for photoproduction and hadroproduction processes) in Ref. [44], and used in Ref. [46] to compute running coupling coefficient functions for DIS.

In order to discuss NLL*x* resummation, we have to carefully specify the choice of factorization scheme. The so-called $Q_0\overline{\text {MS}}$ scheme is often introduced [55, 88, 102, 103], and is preferred to the traditional $\overline{\text {MS}}$ because it gives more stable resummed results. When expanded to fixed order, the scheme-change factor between the two is $\mathcal {O}(\alpha _s^3)$, so NLL*x* resummation in $Q_0\overline{\text {MS}}$ can be matched directly to the usual fixed-order NNLO $\overline{\text {MS}}$ scheme calculation.

### Resummation of DGLAP evolution

Resummed splitting functions take the generic form

2.5 $$\begin{aligned} P_{ij}^{\mathrm{N}^{k}{\mathrm{LO}}+\mathrm{N}^{h}{\mathrm{LL}}x}(x)= P_{ij}^{\mathrm{N}^k{\mathrm{LO}}}(x)+ \Delta _k P_{ij}^{\mathrm{N}^h{\mathrm{LL}}x}(x), \end{aligned}$$

where the first contribution is the splitting function computed to fixed-order *k* (so $k=0,1,2$ for LO, NLO and NNLO) and the second term is the resummed contribution, computed to either LL*x* ($h=0$) or NLL*x* ($h=1$), minus its expansion to the fixed-order *k* to avoid double counting. We note that the splitting functions in the gluon sector ($P_{gg}$ and $P_{gq}$) contain LL*x* and NLL*x* contributions, while in the quark sector ($P_{qg}$ and $P_{qq}$) they only start at NLL*x*. For this reason, there have been attempts to partially extend the resummation to the next logarithmic order (see [118]) which, however, are not considered in this work.

**Fig. 1:**
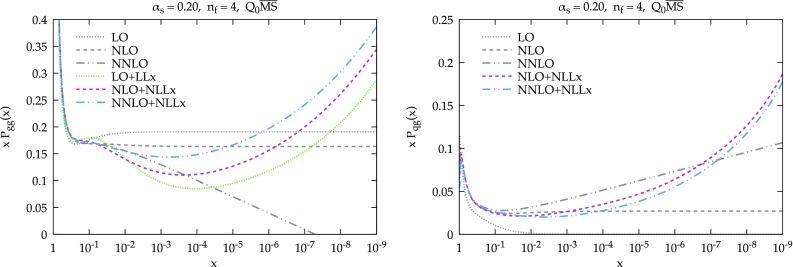
Comparison of the fixed-order gluon–gluon $xP_{gg}(x,\alpha _s)$ (left) and the quark–gluon $xP_{qg}(x,\alpha _s)$ (right) splitting functions with the corresponding LO+LL*x*, $\hbox {NLO+NLL}x$ and $\hbox {NNLO+NLL}x$ results including small-*x* resummation. The comparison is performed at a scale such that $\alpha _s=0.2$ and in the $Q_0\overline{\text {MS}}$ scheme with $n_f=4$ active quark flavors

In Fig. 1 we show a comparison of the fixed-order gluon–gluon $xP_{gg}(x,\alpha _s)$ (left) and the quark–gluon $xP_{qg}(x,\alpha _s)$ (right plot) splitting functions with the resummed counterparts. The comparison is performed in the $Q_0\overline{\text {MS}}$ factorization scheme, with $n_f=4$ active quark flavors and at a small scale such that $\alpha _s=0.2$. We consider LL*x* resummation matched to LO (for the gluon–gluon case), and NLL*x* resummation matched to both NLO and NNLO. All calculations are performed using the HELL (version 2.0) implementation of the ABF construction, and thus incorporate a number of technical improvements which makes the numerical implementation more robust, and allow the matching to NNLO fixed order as well as NLO: a detailed discussion and comparison is given in Refs. [62, 63]. The resummation of small-*x* logarithms is more important at NNLO than at NLO, since at NNLO the fixed-order small-*x* logarithms give rise to perturbative instabilities at small *x*, as visible from a comparison of the NLO and NNLO curves in Fig. 1. Indeed, from the left hand plot, one can immediately see that for moderately small values of *x* NLO gluon evolution is closer to the all-order result at small *x* than NNLO evolution, since for $10^{-6}\lesssim x\lesssim 10^{-3}$ the NLO splitting kernels are closer to the best prediction, $\hbox {NNLO+NLL}x$, than the NNLO ones. Additionally, from the right plot, both resummed results for the gluon-to-quark spitting function are closer to NLO than to NNLO for $10^{-5}\lesssim x\lesssim 10^{-1}$. $\hbox {N}^3\hbox {LO}$ evolution, when available [86, 87], will lead to even more significant instabilities at small *x*, due to the appearance of two extra powers of the small-*x* logarithms (the leading NLO and NNLO logarithms are accidentally zero), and will make the inclusion of small-*x* resummation even more crucial.

To facilitate the use of small-*x* resummation, the HELL code has been interfaced to the public code APFEL [119, 120]. Thanks to this APFEL+HELL interface, it is straightforward to perform the PDF evolution (and the computation of DIS structure functions) with the inclusion of small-*x* resummation effects. Note that APFEL+HELL only implements the so-called “exact” solution of DGLAP evolution, rather than the “truncated” solutions used in ABF (for example in Refs. [44–46]), and nowadays routinely in NNPDF fits, in which subleading corrections are systematically expanded out [72]. For this reason we will use the exact solution throughout in this paper, to facilitate comparison between fixed-order and resummed results. Since the difference between the two solutions becomes smaller and smaller when increasing the perturbative order, this choice does not affect significantly our NNLO(+NLL*x*) results, but care should be taken when comparing the NLO PDFs from those of other NNPDF fits.

**Fig. 2:**
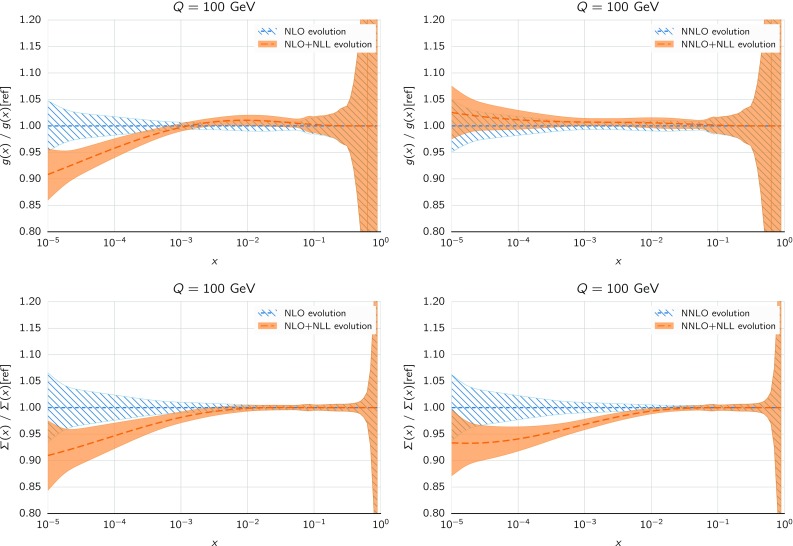
The ratio of the gluon (upper plots) and quark singlet (lower plots) for the evolution from a fixed boundary condition at $Q_0=1.65$ GeV up to $Q=100$ GeV using either fixed-order theory (NLO left, NNLO right) or resummed theory ($\hbox {NLO+NLL}x$ left, $\hbox {NNLO+NLL}x$ right) for the DGLAP evolution. In this specific case, the input boundary condition has been chosen to be NNPDF3.1 NLO (NNLO)

We now investigate the effects induced by evolving the PDFs with resummed splitting kernels as compared to standard fixed-order DGLAP splitting functions. In order to illustrate these effects, we take a given input PDF set as fixed at a low scale $Q_0$, that is, a common boundary condition, and then evolve it upwards using APFEL+HELL with either fixed-order (NLO or NNLO) or resummed ($\hbox {NLO+NLL}x$ or $\hbox {NNLO+NLL}x$) theory. In this way, we can determine what are the main differences induced at high scales by small-*x* resummation in the PDF evolution; we stress, however, that the physical meaning of the resulting comparison is limited, as in a PDF fit with small-*x* resummation the PDFs at low scales, now taken to be equal to their fixed-order counterparts, are likely to change significantly.

The results of this comparison are collected in Fig. 2, where we show the ratio of the gluon (upper plots) and quark singlet (lower plots) as a function of *x* for the evolution from a fixed boundary condition at $Q_0=1.65$ GeV up to $Q=100$ GeV using either (N)NLO fixed-order theory or (N)$\hbox {NLO+NLL}x$ resummed theory for the DGLAP evolution. In this specific case, the input boundary condition has been chosen to be NNPDF3.1 (N)NLO. We observe that the effects of the different PDF evolution settings are negligible at large and medium *x*, but can reach up to a few percent at the smallest values of *x* relevant for the description of the data included in a PDF fit, in particular the HERA structure functions. Specifically, we observe that resummation effects change the NLO evolution quite substantially for both the gluon and the quark singlet, an effect which is reduced at NNLO for the gluon, while it remains of the same size (if not larger) for the quark singlet. Although this study is purely illustrative and by no means predictive, it allows us to conclude that the effect of small-*x* resummation in PDF evolution is in general sizable and will certainly impact the determination of PDFs at small *x*.

**Fig. 3:**
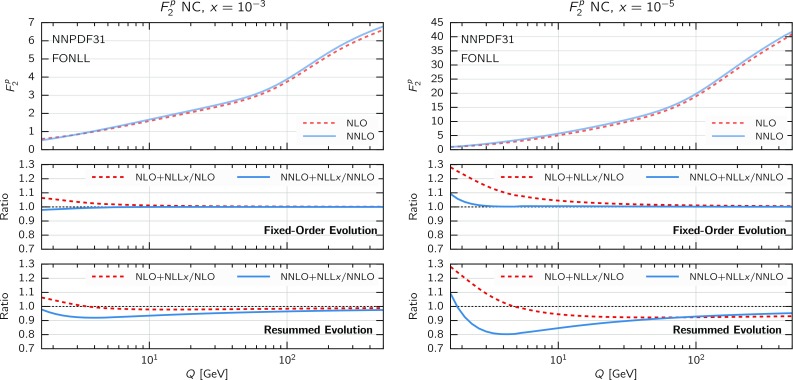
The proton neutral-current (NC) structure function $F_2(x,Q)$ as a function of *Q* for two different values of *x* (left: $x=10^{-3}$; right: $x=10^{-5}$) and using different calculational schemes. In the top panels we show the structure function computed in fixed-order perturbation theory (NLO and NNLO). In the middle and bottom panels we show the ratio of resummed results ($\hbox {NLO+NLL}x$ and $\hbox {NNLO+NLL}x$) to their fixed-order counterparts. In particular, in the middle panel the resummation is included in the coefficient function but not in the evolution, while in the bottom panel we resum both coefficient functions and parton evolution. The input boundary condition at $Q_0=1.65$ GeV has been chosen to be NNPDF3.1 NLO (NNLO), and all calculations are performed with $\alpha _s(m_Z)=0.118$, and a (pole) charm mass $m_c=1.51$ GeV

### Resummation of DIS structure functions

Resummed results for DIS structure functions, including mass effects, have been recently implemented in the public code HELL, version 2.0 [63]. Analogously to Eq. (2.5), resummed and matched results can be written as

2.6 $$\begin{aligned} C_{a,i}^{\mathrm{N}^k{\mathrm{LO}}+\mathrm{NLL}x}(x)= C_{a,i}^{\mathrm{N}^k{\mathrm{LO}}}(x)+ \Delta _k C_{a,i}^{{\mathrm{NLL}}x}(x), \end{aligned}$$

where the index *a* denotes the type of structure function, $a=2, L, 3$, while the index *i* refers to the incoming parton $i=q,g$. Note that in this paper we only consider NLL*x* resummation of the partonic coefficient functions, since in DIS there are no LL*x* contributions. Consistently with the choice made for the evolution, we work in the $Q_0\overline{\text {MS}}$ scheme.

A consistent PDF fit which spans several orders of magnitude in $Q^2$ further requires us to consider a different number of active quark flavors at different energies, to account for potentially large collinear logarithms due to massive quarks. When crossing the threshold of a given heavy quark, matching conditions which relate the PDFs above and below threshold are needed. These matching conditions also contain small-*x* logarithmic enhancements, which one can consistently resum. As for DIS coefficient functions, the matching conditions are NLL*x*, and their resummation, as well as the resummation of the massive coefficient functions [63, 110] is available in HELL 2.0. These last ingredients make it straightforward to implement a resummation of the FONLL variable flavor number scheme [121] used in the NNPDF fits.

A careful treatment of charm is essential when addressing the impact of small-*x* resummation on DIS structure functions, since the kinematic region where resummation is expected to be important (small *x* and low $Q^2$) is rather close to the charm threshold. We thus fit the initial charm distribution, as in Ref. [78]. The FONLL scheme can be readily extended to fitted charm, in the process receiving an extra contribution [122], denoted $\Delta _{\mathrm{IC}}$, which is currently known only at $\mathcal {O}(\alpha _s)$ [123, 124]. When $\Delta _{\mathrm{IC}}$ is included, the phenomenological damping adopted in the original FONLL formulation to smooth the transition to the regime in which collinear logarithms are resummed does not have any effect [122, 124], and is therefore omitted. Since the $\mathcal {O}(\alpha _s)$ $\Delta _{\mathrm{IC}}$ contribution is then a small correction, we expect the NNLO ($\mathcal {O}(\alpha _s^2)$) and small-*x* resummation corrections to $\Delta _{\mathrm{IC}}$ to be practically insignificant (see Ref. [78] for a detailed discussion of this issue).

To obtain a first qualitative estimate of the impact of small-*x* resummation in the DIS structure functions, we can compare theoretical predictions at (N)NLO with predictions that include resummation. To disentangle the effect of resummation on PDF evolution from that in the coefficient functions in the $Q_0\overline{\text {MS}}$ scheme, we take into account the effect of resummation in two steps. First, we compute structure functions with the same (fixed-order) input PDFs and include small-*x* resummation in the coefficient functions only. As a second step, we include resummation also in the DGLAP evolution, using a fixed input PDF boundary condition at a small scale $Q_0=1.65$ GeV, as previously done in Fig. 2. Since, as already noticed, the use of a fixed boundary condition at a small scale is not particularly physical, these results should be interpreted with care.

**Fig. 4:**
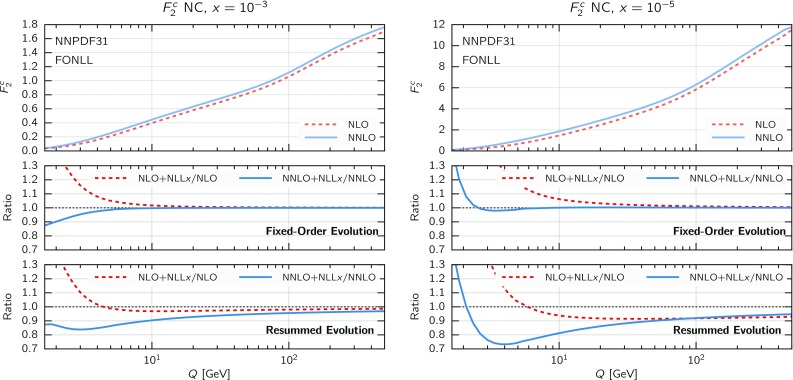
Same as Fig. 3 for $F_2^c(x,Q)$, the charm component of the structure function $F_2(x,Q)$

**Fig. 5:**
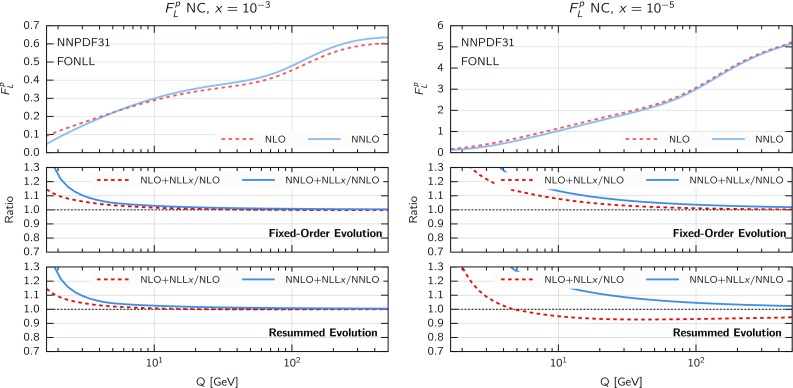
Same as Fig. 3 for the proton longitudinal structure function $F_L(x,Q)$

The proton structure function $F_2(x,Q)$ in neutral-current (NC) DIS is shown in Fig. 3 as a function of *Q* for two values of *x*, one moderate ($x=10^{-3}$, left plot) and one small ($x=10^{-5}$, right plot). The upper panel of each plot shows the NLO and NNLO results. The middle panel shows the ratio of resummed (N)$\hbox {NLO+NLL}x$ theory over the fixed-order (N)NLO results, including resummation only in coefficient functions. The lower panel, instead, shows the same ratio but with resummation included also in PDF evolution. In all cases, we take the NNPDF3.1 boundary condition at (N)NLO at $Q_0=1.65$ GeV. As mentioned above, heavy-quark mass effects are included using the FONLL-B (C) scheme [121, 122, 124] for the NLO (NNLO) calculations, supplemented with small-*x* resummed contribution for the (N)$\hbox {NLO+NLL}x$ as described in Ref. [63].

The comparison in Fig. 3 is interesting from several points of view. First of all, we observe that when resummation is included only in the coefficient functions its effect is rather mild, almost negligible when matched to NNLO, even at rather small *x* and at low scales. On the other hand, when including resummation in the PDF evolution, the situation changes. In this case, we note that the differences between fixed-order and resummation are larger, thus showing that in $F_2$ much of the impact of small-*x* resummation arises from the PDF evolution. Moreover, the effects are always greater at NNLO than at NLO: at NNLO, effects of small-*x* resummation can reach 10 percent already for $x\simeq 10^{-3}$, and 20 percent for $x\simeq 10^{-5}$. This discussion suggests that at the level of PDF fits we expect little differences between the fixed-order and resummed cases at NLO, but more significant differences at NNLO.

Next, in Fig. 4 we show the same comparison as in Fig. 3 but now for $F_2^c(x,Q)$, the charm component of the proton structure function $F_2(x,Q)$. By comparing Figs. 3 and 4 we observe that the impact of small-*x* resummation for inclusive and charm structure functions is similar, except just above the charm threshold where the effects of the resummation in the charm coefficient function can be substantial. From this comparison, we see the importance of a careful treatment of mass effects close to the charm threshold, since these can change the size of the effect of small-*x* resummation.

Finally, in Fig. 5 we show the corresponding comparison but this time for the longitudinal structure function $F_L(x,Q)$ in neutral-current DIS. Here we find that resummation effects in the coefficient functions only are substantially larger than in $F_2$, and are now larger when matching resummation to NNLO than to NLO. When resummation is included also in PDF evolution, the overall effect of resummation on $F_L$ is somewhat reduced at NLO, thus showing some sort of compensation of the effects in PDF evolution and in partonic coefficient functions, while it is enlarged at NNLO, which now reaches about a $30\%$ deviation at $x=10^{-5}$ at small $Q\sim 5$ GeV. The global pattern is similar to $F_2$, with differences smaller at NLO and more significant at NNLO, though overall effect is somewhat bigger, consistently with the fact that $F_L$ is singlet dominated. Given that $F_L$ contributes to the measured reduced cross-sections $\sigma _{r,\mathrm NC}$ at high *y*, which for the HERA kinematics corresponds to small *x* and $Q^2$, this effect should be relevant for PDF fits.

## Fitting strategy

In this section we discuss the settings of the NNPDF3.1 fits with small-*x* resummation, as well as of their fixed-order counterparts, which are used as baseline comparisons. In the following, we will denote these fits as NNPDF3.1sx, each of them consisting of $N_{\mathrm{rep}}=100$ Monte Carlo replicas. We briefly present the input dataset, and review the theoretical treatment of the deep-inelastic and hadronic data used in the fit. We also discuss the strategy adopted for choosing appropriate kinematic cuts for both DIS and hadronic processes.

### Fit settings

The settings of the fits described in this work follow closely those of the recent NNPDF3.1 global analysis [79]. In particular, the same input dataset is used, which includes fixed-target [125–132] and HERA [68] DIS inclusive structure functions; charm and bottom cross-sections from HERA [133]; fixed-target Drell–Yan (DY) production [134–137]; gauge boson and inclusive jet production from the Tevatron [138–142]; and electroweak boson production, inclusive jet, *Z* $p_T$ distributions, and $t\bar{t}$ total and differential cross-sections from ATLAS [143–157], CMS [158–169] and LHCb [170–174] at $\sqrt{s}=7$ and 8 TeV.

As in the NNPDF3.1 analysis, the charm PDF is fitted alongside the light quark PDFs [78], rather than being generated entirely from perturbative evolution off gluons and light quarks. As usual in NNPDF, we use heavy-quark pole masses [175], and the charm quark pole mass is taken to be $m_c=1.51$ GeV. In all the results presented here we take $\alpha _s(m_Z) = 0.118$.

The initial scale $Q_0$ at which PDFs are parametrized is chosen to be $Q_0=1.64$ GeV, i.e. $Q_0^2=2.69$ GeV$^2$, which is slightly smaller than the initial scale adopted in the NNPDF3.1 analysis, namely $Q_0=1.65$ GeV. The main motivation for this choice of initial scale is to be able to include the $Q^2=2.7$ GeV$^2$ bin in the HERA inclusive structure function data [68], which is expected to be particularly sensitive to the effects of small-*x* resummation, and that was excluded from NNPDF3.1. At the same time, the initial scale cannot be too low, to avoid entering a region in which $\alpha _s$ is too large and the numerical reliability of the small-*x* resummation implemented in the HELL code would be lost.^2^

In this work we have produced fits at fixed-order NLO and NNLO accuracy and corresponding resummed fits at $\hbox {NLO+NLL}x$ and $\hbox {NNLO+NLL}x$ accuracy. In the resummed fits, small-*x* resummation is included both in the solution of the evolution equations and in the deep-inelastic coefficient functions as discussed in Sect. 2. Heavy-quark mass effects are accounted for using the FONLL-B and FONLL-C general-mass scheme [121, 122, 124] for the NLO and NNLO fits, respectively, modified to include small-*x* resummation effects when $\hbox {NLO+NLL}x$ and $\hbox {NNLO+NLL}x$ theory is used as previously described.

Theoretical predictions for the Drell–Yan fixed-target and the hadron collider (Tevatron and LHC) cross-sections are obtained using fixed-order or resummed DGLAP evolution for (N)NLO and (N)$\hbox {NLO+NLL}x$ fits, respectively, but with their partonic cross-sections always evaluated at the corresponding fixed order. This approximation is due to the fact that the implementation of hadronic processes in HELL is still work in progress. To account for this limitation, we cut all data in kinematic regions where small-*x* corrections are expected to be significant, as explained in Sect. 3.2 below.

The settings for the evaluation of the hadronic hard-scattering matrix elements are the same as in NNPDF3.1, namely we use fast NLO calculations as generated by APPLgrid [176] and FastNLO [177] tables, which are combined before the fit with the DGLAP evolution kernels by means of the APFELgrid interface [178]. For the NNLO fits, NNLO/NLO point-by-point *K*-factors are used [79] using specific codes for each process: we use the code of [179, 180] for $t\bar{t}$ differential distributions [181]; for the *Z* $p_T$ distributions we use the calculation of [182, 183]; for Drell–Yan production we use FEWZ [184]; while jet cross-sections are treated using NLO matrix elements supplemented by scale variation as additional theory systematics.

For comparison purposes, we have also produced DIS-only fits for which small-*x* resummation is included in both evolution and coefficient functions for all data points included in the fit. That is, in such fit, fully consistent small-*x* resummed theory is used for the entire dataset. Moreover, while PDF uncertainties are of course much larger due to the lack of hadronic data, the constraints from the HERA structure functions are still the dominant ones in the small-*x* region. The comparison between the global and DIS-only NNPDF3.1sx fits is discussed in Sect. 4.2.1.

### Kinematic cuts

In the NNPDF3.1sx analysis, we apply the same experimental cuts as those of the NNPDF3.1 fit [79] with two main differences. First, as discussed above, the lower $Q^2$ cut is reduced from $Q^2_{\mathrm{min}}=3.49$ GeV$^2$ in NNPDF3.1 to $Q^2_{\mathrm{min}}=2.69$ GeV$^2$ here. Thanks to this lower cut, we can now include a further bin of the HERA inclusive cross-section data, specifically the one with $Q^2=2.7$ GeV$^2$. In turn, this allows us to slightly extend the kinematic coverage of the small-*x* region, from $x_{\mathrm{min}} \simeq 4.6 \times 10^{-5}$ before, down to $x_{\mathrm{min}} \simeq 3 \times 10^{-5}$ now. This lower cut also affects a handful of points at low $Q^2$ (although at larger values of *x*) of other fixed-target DIS experiments, which are therefore also included in the NNPDF3.1sx fits but not in NNPDF3.1. The cut on $W^2\ge 12.5$ GeV$^2$ remains the same.

Moreover, no additional cuts are applied to the HERA charm-production cross-sections as compared to the inclusive structure functions. This was not the case in NNPDF3.1, where some points at small-*x* and $Q^2$ were excluded in the NNLO fit, specifically those with $Q^2\le 8$ GeV$^2$. We have explicitly verified that the inclusion of these extra points does not affect the resulting PDFs, though the $\chi ^2$ of the $F_2^c$ data becomes somewhat worse at NNLO. Taking into account these two differences, from HERA we fit 1162 points for the inclusive structure functions and 47 points for the $F_2^c$ data, to be compared with 1145 (1145) and 47 (37) in NNPDF3.1 NLO (NNLO), respectively. The number of data points $N_{\mathrm{dat}}$ for each of the DIS experiments included in NNPDF3.1sx is collected in Table 1.

**Table 1:** The number of data points $N_{\mathrm{dat}}$ for each of the DIS experiments included in NNPDF3.1sx

Experiment	$N_{\mathrm{dat}}$
NMC	367
SLAC	80
BCDMS	581
CHORUS	886
NuTeV dimuon	79
HERA I+II incl. NC	1081
HERA I+II incl. CC	81
HERA $\sigma _c^{\mathrm{NC}}$	47
HERA $F_2^b$	29
Total	3231

The second main difference with respect to the NNPDF3.1 kinematic cuts is related to hadronic data. As already discussed, for hadronic processes small-*x* resummation effects are included only in PDF evolution but not in the partonic cross-sections. Therefore, in order to avoid biasing the fit results, in the NNPDF3.1sx fits we include only those hadronic data for which the effects of small-*x* resummation on the coefficient function can be assumed to be negligible.

**Fig. 6:**
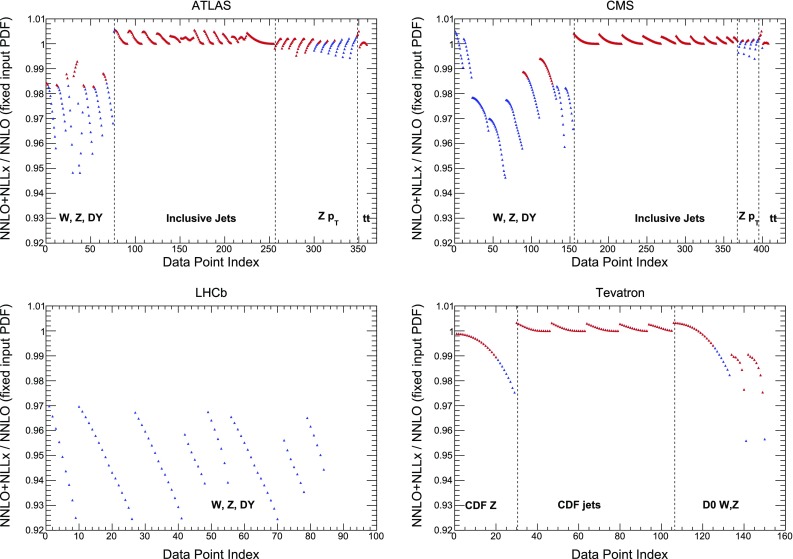
The ratio of hadronic cross-sections included NNPDF3.1 computed using a fixed input PDF at $Q_0=1.65$ GeV (in this case NNPDF3.1 NNLO) using either $\hbox {NNLO+NLL}x$ or NNLO theory for PDF evolution, always with NNLO partonic cross-sections. We show the results for ATLAS, CMS, LHCb, and the Tevatron, indicating the division of each experiment into families of processes. The empty blue triangles indicate those data points that are excluded from the NNPDF3.1sx fits with the default cut $H_{\mathrm{cut}}=0.6$, while the filled red ones indicate the points that satisfy the condition Eq. (3.2)

Quantifying the impact of small-*x* resummation on the partonic coefficient functions would require the knowledge of such resummation. Therefore, in order to estimate the region of sensitivity to small-*x* logarithms, we resort to a more qualitative argument. The foundation of this argument is the observation that in a generic factorization scheme large logarithms appear both in the partonic coefficient functions and in the partonic evolution factors; in general, resummation corrections are thus expected to have a similar size both in the evolution and in the coefficient functions. This naive expectation is indeed confirmed by explicit calculations of hadronic resummed cross-sections [44, 185], where it was found that the most common situation is a partial cancellation between the resummation corrections from evolution and those in the partonic cross-section. It follows that estimates based on the corrections due to resummed evolution alone will probably be conservative, in the sense that they will over-estimate the total resummation correction to the hadronic cross-section.

In order to implement these cuts, we first introduce a parametrization of the resummation region in the $(x,Q^2)$ plane. Small-*x* logarithmic corrections should in principle be resummed when $\alpha _s(Q^2) \ln 1/x$ approaches unity, since the fixed-order perturbative expansion then breaks down. We thus define our kinematic cut to the hadronic data in the NNPDF3.1sx fits such as to removes those data points for which

3.1 $$\begin{aligned} \alpha _s(Q^2) \ln {{1}\over {x}} \ge H_{\mathrm{cut}}, \end{aligned}$$

where $H_{\mathrm{cut}}\lesssim 1$ is a fixed parameter: the smaller $H_{\mathrm{cut}}$, the more data are removed. Assuming one-loop running for the strong coupling constant (which is enough for our purposes), Eq. (3.1) can instead be expressed as

3.2 $$\begin{aligned} \ln {{1}\over {x}}\ge \beta _0 H_{\mathrm{cut}} \ln {{Q^2}\over {\Lambda ^2}}, \end{aligned}$$

where $\Lambda \simeq 88$ MeV is the QCD Landau pole for $n_f=5$, and $\beta _0\simeq 0.61$. Thus the cut is a straight line in the plane of $\ln {{1}\over {x}}$ and $\ln {{Q^2}\over {\Lambda ^2}}$, with gradient $\beta _0 H_{\mathrm{cut}}$.

**Fig. 7:**
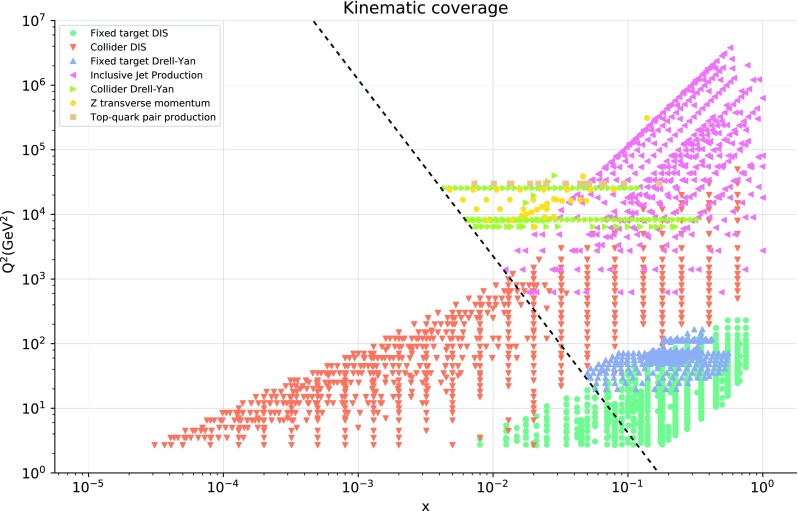
The kinematic coverage in the $(x,Q^2)$ plane of the data included in the NNPDF3.1sx fit with the default value of the kinematic cut to the hadronic data, $H_{\mathrm{cut}}=0.6$. The diagonal line indicates the value of the cut Eq. (3.2), below which the hadronic data is excluded from the fit. For hadronic processes, the LO kinematics have been used to determine the $(x,Q^2)$ values associated to each data bin

Note that the variable *x* used in the definition of the cut, Eq. (3.1), can in general only be related to the final-state kinematic variables of hadronic observables by assuming leading-order kinematics. To see how this works in practice, consider for example weak gauge boson production: then $Q^2=M_V^2$, and for fixed $\sqrt{s}$ the cut translates into a maximum rapidity

3.3 $$\begin{aligned} y_{\mathrm{max}}= \ln {{M_V}\over {\sqrt{s}}}+\beta _0H_{\mathrm{cut}}\ln {{M_V^2}\over {\Lambda ^2}}. \end{aligned}$$

Thus in the case of *W* boson production at $\sqrt{s}=7$ TeV, a cut of the form of Eq. (3.2) with $H_{\mathrm{cut}}=0.5$ (0.7) would imply that cross-sections with rapidities above $y_{\mathrm{max}} \simeq 0.3~(1.3)$ would be excluded from the fit. In this case, the first (tighter) cut excludes all the LHC gauge boson production data except for a handful of points from the ATLAS and CMS measurements in the most central rapidity region. The second (looser) cut instead allows one to include most of the ATLAS and CMS gauge boson production data. However, the LHCb measurements are removed altogether for both values of the cut, highlighting the sensitivity of forward *W*, *Z* production data to the small-*x* region.

It remains to determine the optimal value of $H_{\mathrm{cut}}$, in a way that minimizes at the same time the amount of information lost from the dataset reduction, but also the possible theoretical bias due to the missing small-*x* resummed coefficient functions. In this work we will present results with three different values, namely $H_{\mathrm{cut}}=0.5, 0.6$ and 0.7. In Sect. 4 we will motivate the choice of $H_{\mathrm{cut}}=0.6$ as our default value, and show explicitly how the main findings on this work are independent of the specific value of $H_{\mathrm{cut}}$ adopted.

Here we attempt to provide an a priori argument to justify our choice by estimating the size of the resummation corrections through a comparison of the results obtained with fixed-order and resummed parton evolution. Specifically, we take a fixed input PDF set (NNPDF3.1 NNLO) at $Q_0=1.65$ GeV and evolve it using either NNLO or $\hbox {NNLO+NLL}x$ theory, and then compute the convolution with fixed-order partonic coefficient functions. The comparison is represented in Fig. 6, where we show the ratio of hadronic cross-sections computed using $\hbox {NNLO+NLL}x$ evolution over those computed using NNLO evolution. We show the results for ATLAS, CMS, LHCb, and the Tevatron data points included in NNPDF3.1, indicating the division of each experiment into families of processes. From this comparison, we see that the effects of small-*x* resummation are likely to be significant only for the *W* and *Z* Drell–Yan data, where they could be as large as up to $\sim 5\%$ for ATLAS and CMS, and up to $\sim 8\%$ for the forward LHCb measurements, while they are most likely negligible for all other collider processes, such as jets, the *Z* $p_T$, and top-quark pair production. Given that the collider DY data have rather small experimental uncertainties, of the order of a few percent or even smaller, we should ensure that we cut data where the effects of small-*x* resummation could be larger than $\sim 2\%$ (to be conservative). We see from Fig. 6 that this is indeed achieved with the default value of $H_{\mathrm{cut}}=0.6$: for the included points, differences are always smaller than this threshold.

To summarize this discussion of the kinematic cuts in the NNPDF3.1sx fits, we show in Fig. 7 the kinematic coverage in the $(x,Q^2)$ plane of the data included in the present analysis, for the default value $H_{\mathrm{cut}}=0.6$ of the cut to the hadronic data. As mentioned above, for hadronic processes the LO kinematics have been used to determine the values of *x* and $Q^2$ associated to each data bin. The diagonal line indicates the region below which the cut defined in Eq. (3.2) removes hadronic data. As a consequence of the kinematic cuts, the hadronic dataset is restricted to the large-$Q^2$ and medium- and large-*x* region.

In Table 2 we show the number of data points for the hadronic data in the NNPDF3.1sx NNLO fits for with $H_{\mathrm{cut}}=0.5,0.6$ and 0.7. The number in brackets corresponds to the values for the NLO fits, since the kinematic cuts of the NNPDF3.1 fits [79] are slightly different at NLO and at NNLO. The main effect of the $H_{\mathrm{cut}}$ is on the Drell–Yan prediction measurements from ATLAS and CMS, which in turn affects the quark and antiquark flavor separation, and the *Z* $p_T$ distributions, which provide information on the gluon. On the other hand, the inclusive jet and top-quark pair production data, which are mostly sensitive to the large-*x* region, are essentially unaffected by the cut. For completeness, we also provide the values of $N_{\mathrm{dat}}$ when no cut is applied at all ($H_{\mathrm{cut}}=\infty$). In the latter case, the fit also includes 85 (93) LHCb experimental points at NNLO (NLO).

**Table 2:** The number of data points $N_{\mathrm{dat}}$ for each of the hadronic experiments included in the NNLO NNPDF3.1sx global fits for different values of $H_{\mathrm{cut}}=0.5,0.6$ and 0.7, with the default value being $H_{\mathrm{cut}}=0.6$. The number in brackets corresponds to the values for the NLO fits, if different from the NNLO value. For completeness, we also show $N_{\mathrm{dat}}$ when the $H_{\mathrm{cut}}$ is not applied ($H_{\mathrm{cut}}=\infty$). The last row indicates the total number of hadronic data points included in the fit for each value of the cut

Experiment	$N_{\mathrm{dat}}$			
	$H_{\mathrm{cut}}=0.5$	$H_{\mathrm{cut}}=0.6$	$H_{\mathrm{cut}}=0.7$	$H_{\mathrm{cut}}=\infty$
DY E866 $\sigma ^d_{\mathrm{DY}}/\sigma ^p_{\mathrm{DY}}$	11	13	14	15
DY E886 $\sigma ^p$	55	75	87	89
DY E605 $\sigma ^p$	85	85	85	85
CDF *Z* rap	12	20	29	29
CDF Run II $k_t$ jets	76	76	76	76
D0 *Z* rap	12	20	28	28
D0 $W\rightarrow e\nu$ asy	4	7 (8)	8 (12)	8 (13)
D0 $W\rightarrow \mu \nu$ asy	4	8 (9)	9 (10)	9 (10)
ATLAS total	230	258	294	354
ATLAS *W*, *Z* 7 TeV 2010	0	6	16	30
ATLAS HM DY 7 TeV	5	5	5	5
ATLAS *W*, *Z* 7 TeV 2011	0	8	20	34
ATLAS jets 2010 7 TeV	81	86	89	90
ATLAS jets 2.76 TeV	56	59	59	59
ATLAS jets 2011 7 TeV	31	31	31	31
ATLAS *Z* $p_T$ 8 TeV $(p_T^{ll},M_{ll})$	44	44	44	44
ATLAS *Z* $p_T$ 8 TeV $(p_T^{ll},y_{ll})$	0	6	17	48
ATLAS $\sigma _{tt}^{tot}$	3	3	3	3
ATLAS $t\bar{t}$ rap	10	10	10	10
CMS total	234	259	316	409 (387)
CMS *W* asy 840 pb	0	0	7	11
CMS *W* asy 4.7 fb	0	0	7	11
CMS *W* rap 8 TeV	0	0	12	22
CMS Drell–Yan 2D 2011	8	24	44	110 (88)
CMS jets 7 TeV 2011	133	133	133	133
CMS jets 2.76 TeV	81	81	81	81
CMS *Z* $p_T$ 8 TeV $(p_T^{ll},y_{ll})$	3	10	19	28
CMS $\sigma _{tt}^{tot}$	3	3	3	3
CMS $t\bar{t}$ rap	6	8	10	10
LHCb total	0	0	0	85 (93)
LHCb *Z* rapidity 940 pb	0	0	0	9
LHCb $Z\rightarrow ee$ rapidity 2 fb	0	0	0	17
LHCb $W, Z \rightarrow \mu$ 7 TeV	0	0	0	29 (33)
LHCb $W, Z \rightarrow \mu$ 8 TeV	0	0	0	30 (34)
Total	723	821 (823)	946 (951)	1187 (1179)

## Parton distributions with small-*x* resummation

In this section we present the main results of this work, namely the NNPDF3.1sx fits including the effects of small-*x* resummation. We will present first the DIS-only fits and then the global fits, based on the dataset described in Sect. 3. Unless otherwise specified, for the global fits we will use the default cut $H_{\mathrm{cut}}=0.6$ for the hadronic data.

In the following, we will first discuss the DIS-only fits, showing how small-*x* resummation improves the fit quality and affects the shape of the PDFs. We then move to the global fits, and compare them to the DIS-only ones. We find that the qualitative results are similar, though PDF uncertainties are reduced. We show the impact of resummation on the PDFs, and study the dependence on the cut used to remove the hadronic data potentially sensitive to small-*x* logarithms and for which we do not yet include resummation. We show how our default choice for $H_{\mathrm{cut}}$ does not bias the fit, and still allows us to determine PDFs whose uncertainties are competitive with those of NNPDF3.1. We discuss in detail the role of the additional low-$Q^2$ HERA bin that we include in this fit for the first time, and how small-*x* resummed theory is able to fit it satisfactorily.

**Table 3:** The values of $\chi ^2/N_{\mathrm{dat}}$ for the total and the individual datasets included in the DIS-only NNPDF3.1sx NLO, $\hbox {NLO+NLL}x$, NNLO and $\hbox {NNLO+NLL}x$ fits. The number of data points $N_{\mathrm{dat}}$ for each experiment is indicated in Table 1. In addition, we also indicate the absolute difference $\Delta \chi ^2$ between the resummed and fixed-order results, Eq. (4.1). We indicate with a dash the case $|\Delta \chi ^2| < 0.5$

	$\chi ^2/N_{\mathrm{dat}}$		$\Delta \chi ^2$	$\chi ^2/N_{\mathrm{dat}}$		$\Delta \chi ^2$
	NLO	$\hbox {NLO+NLL}x$		NNLO	$\hbox {NNLO+NLL}x$	
NMC	1.31	1.32	$+5$	1.31	1.32	$+4$
SLAC	1.25	1.28	$+2$	1.12	1.02	$-8$
BCDMS	1.15	1.16	$$ +7$$	1.13	1.16	$$+14$$
CHORUS	1.00	1.01	$$+9 $$	1.00	1.03	$$+26 $$
NuTeV dimuon	0.66	0.56	$-8$	0.80	0.75	$-4$
HERA I+II incl. NC	1.13	1.13	$+6$	1.16	1.12	$-47$
HERA I+II incl. CC	1.11	1.09	$-1$	1.11	1.11	–
HERA $\sigma _c^{\mathrm{NC}}$	1.44	1.35	$-5$	2.45	1.24	$-57$
HERA $F_2^b$	1.06	1.14	$+2$	1.12	1.17	$+2$
Total	1.113	1.119	$+17$	1.139	1.117	$-70$

We will further inspect the improved description of the HERA data in Sect. 5, where we will perform a number of diagnostic studies aimed at quantifying the onset of BFKL dynamics in the inclusive HERA structure functions.

### DIS-only fits

Let us start our discussion by considering the DIS-only fits, in which we include all the DIS data from fixed-target and collider experiments described in Sect. 3. For all these data, we have a complete theoretical description at resummed level, thus allowing us to perform a fully consistent small-*x* resummed fit. First of all, in Table 3 we collect the $\chi ^2/N_{\mathrm{dat}}$ values for the total and individual datasets computed with the PDFs fitted using NLO, $\hbox {NLO+NLL}x$, NNLO and $\hbox {NNLO+NLL}x$ theory. The $\chi ^2$ values are computed using the experimental definition of the covariance matrix, while the $t_0$ definition [186] was instead used during the fits, as customary in the NNPDF analyses. In addition, we also show the difference in $\chi ^2$ between the resummed and fixed-order results,

4.1 $$\begin{aligned} \Delta \chi ^2_{\mathrm{(N)NLO}} \equiv \chi ^2_{{\mathrm{(N)NLO+NLL}}x}-\chi ^2_{\mathrm{(N)NLO}}, \end{aligned}$$

which is useful to gauge how statistically significant are the differences between the fixed-order and resummed results for each experiment.

We immediately observe that the $\hbox {NNLO+NLL}x$ fit has a total $\chi ^2/N_{\mathrm{dat}}$ that improves markedly with respect to the NNLO result, which instead gives the highest value of $\chi ^2/N_{\mathrm{dat}}$. The total $\chi ^2/N_{\mathrm{dat}}$ is essentially the same in the NLO, $\hbox {NLO+NLL}x$, and $\hbox {NNLO+NLL}x$ fits. As illustrated by the $\Delta \chi ^2$ values of Table 3, the bulk of the difference in the fit quality between the NNLO and $\hbox {NNLO+NLL}x$ fits arises from the HERA inclusive neutral-current and charm datasets, which probe the smallest values of *x*, and whose $\chi ^2/N_{\mathrm{dat}}$ decrease from 1.16 to 1.12 ($\Delta \chi ^2=-47$) and from 2.45 to 1.24 ($\Delta \chi ^2=-57$), respectively.

**Fig. 8:**
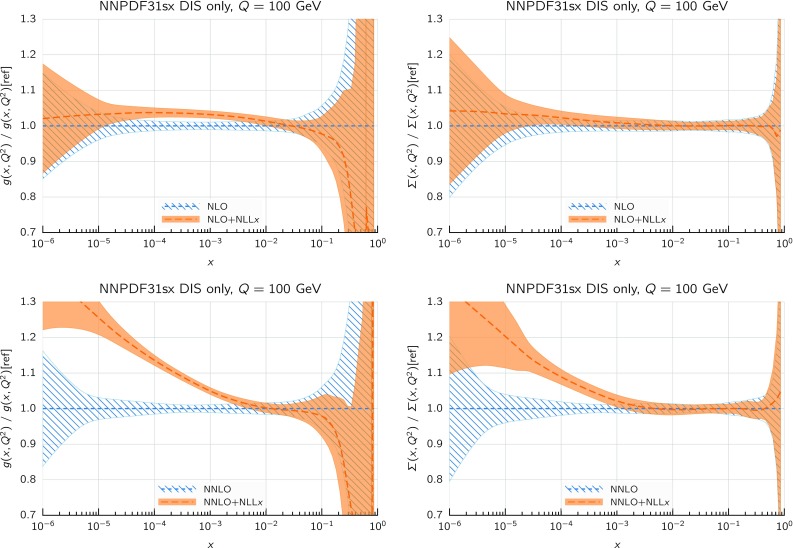
Comparison between the gluon (left) and the total quark singlet (right plots) from the NLO and $\hbox {NLO+NLL}x$ (upper plots) and from the NNLO and $\hbox {NNLO+NLL}x$ DIS-only fits (lower plots). The comparison is performed at $Q=100$ GeV, normalized to the central value of the corresponding fixed-order fit, and the bands indicate the 68% confidence level PDF uncertainties

We note that the $\chi ^2/N_{\mathrm{dat}}$ of the charm dataset is rather high at NNLO. In fact, the description of the charm data can be rather sensitive to the details of the heavy quark scheme. For instance, we can set to zero the $\Delta _{\mathrm{IC}}$ term discussed in Sect. 2.3, thus allowing the inclusion of a phenomenologically induced damping factor which has the role of suppressing formally subleading terms numerically relevant at scales close to the charm threshold (see [121, 122, 124]).^3^ When the damping is included, we find that recomputing the $\chi ^2/N_{\mathrm{dat}}$ of the charm dataset it becomes 1.10 at NNLO. On the other hand, the quality of resummed theory is very stable with respect to such a variation, and the $\chi ^2/N_{\mathrm{dat}}$ of the charm data becomes 1.23 ($\Delta \chi ^2=+6$). The rather high value of the charm data $\chi ^2$ at NNLO with our default settings is mostly driven by a poor description of the low-*x* and low-$Q^2$ bins. Indeed, if we restrict our attention to the region which survives the more conservative cut used in NNPDF3.1 ($Q^2 \ge 8$ GeV$^2$ for the HERA charm data), we obtain $\chi ^2/N_{\mathrm{dat}}=1.38$ at NNLO and 1.35 at $\hbox {NNLO+NLL}x$ ($\Delta \chi ^2=-1$) using our default settings. The low-$Q^2$ region is somewhat affected by how the subleading terms are treated – ultimately, this choice is driven by phenomenological reasons, and therefore it is possible that by tuning them one may achieve a satisfactory description of the data at NNLO, for instance by mimicking a perturbative behavior^4^; however, the same choice may be suboptimal at the resummed level. Since at NLO(+NLL*x*) and with FONLL-B we achieve a satisfactory description of the charm data for all 47 points both at fixed-order and at resummed level, here we shall use the same theory settings of the NNPDF3.1 paper, and interpret the more marked dependence on the subleading terms as a limitation of the fixed-order theory at NNLO.

We further observe that the description of the fixed-target DIS experiments, sensitive to the medium and small-*x* region, is not significantly affected by the inclusion of small-*x* resummation, giving us confidence that the resummed and matched predictions reduce to their fixed-order counterpart where they should. The only exception is the slight decrease in fit quality between the NNLO and $\hbox {NNLO+NLL}x$ fits for BCDMS and CHORUS ($\Delta \chi ^2=+14$ and $+26$, respectively). As we will show in the next section, most of these differences go away once the collider dataset is included in the global fit, stabilizing the large *x* PDFs.

Another interesting result from Table 3 is that the effect of resummation is instead much less marked at NLO. Indeed, the NLO and $\hbox {NLO+NLL}x$ fits have very similar $\chi ^2/N_{\mathrm{dat}}$: in particular the $\chi ^2$ change of the HERA inclusive (charm) dataset is rather small, $\Delta \chi ^2=+6\,(-5)$. This is again not surprising, as the whole point of resummation is to cure instabilities in the fixed-order perturbative expansion, by removing the large logarithms causing the instability and replacing them with all-order results. Thus the resummation is more important at NNLO than at NLO, and indeed would probably be yet more important at the next perturbative order ($\hbox {N}^3\hbox {LO}$).

**Fig. 9:**
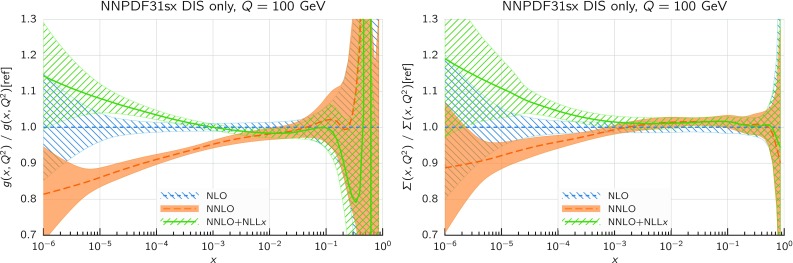
Comparison between the gluon (left) and quark singlet (right plot) PDFs in the NNPDF3.1sx DIS-only fits using NLO, NNLO, and $\hbox {NNLO+NLL}x$ theory at $Q=100$ GeV, normalized to the central value of the former

We can see this result more clearly by considering the resulting fitted PDFs and their uncertainties. In Fig. 8 we show the ratio between the gluon (left) and the total quark singlet (right) at $Q=100$ GeV in the $\hbox {NLO+NLL}x$ fit as compared to the NLO baseline (upper plots) and in the $\hbox {NNLO+NLL}x$ fit as compared to the NNLO baseline (lower plots). In this comparison, as well as in subsequent PDF plots, the bands represent the 68% confidence level PDF uncertainty. Consider first the $\hbox {NLO+NLL}x$ fit. Here the resummation has a moderate effect: the resummed gluon PDF is somewhat enhanced between $x=10^{-5}$ and $x=10^{-2}$, with the PDF uncertainty bands only partially overlapping, whilst the shift in central values for the singlet is well within the PDF uncertainties. This remains true down to the smallest values of *x*: even for values as small as $x\simeq 10^{-6}$ the shifts of the central value of the singlet and the gluon PDF due to the resummation are less than 10%. This is a consequence of the fact that, as discussed in Sect. 2, NLO theory is a reasonably good approximation to the fully resummed result at small *x*, and any differences are such that can be reabsorbed into small changes in the gluon PDF.

The situation is rather different at $\hbox {NNLO+NLL}x$. In this case, we see that starting from $x\lesssim 10^{-3}$ the resummed gluons and quarks are systematically higher than in the baseline NNLO fit, by an amount which ranges from 10% for $x\sim 10^{-4}$ up to 20% for $x\sim 10^{-5}$ (though note that in this analysis there are no experimental constraints for $x\lesssim 3\times 10^{-5}$). The shifts outside central values are significantly outside the PDF uncertainty bands, yet result in an improvement in the quality of the fit.

Note that we are performing these comparisons at the electroweak scale $Q\sim 100$ GeV, where there are no DIS data and where the effect of resummed evolution is combined with the change of the fitted PDFs at low scales. This has the advantage of showing that several observables at the LHC characterized by electroweak scales are likely to be sensitive to small-*x* resummation through the PDFs, particularly when measurements can be performed at high rapidities. Therefore, for such observables, the use of small-*x* resummed PDFs (and coefficient functions) is probably going to be necessary in order to obtain reliable theoretical predictions.

**Fig. 10:**
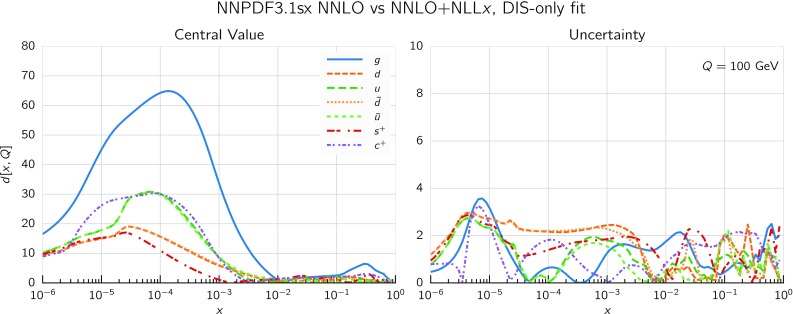
The statistical distances between the central values (left) and the PDF uncertainties (right plot) of the NNPDF3.1sx NNLO and $\hbox {NNLO+NLL}x$ fits at $Q=100$ GeV in the flavor basis

**Fig. 11:**
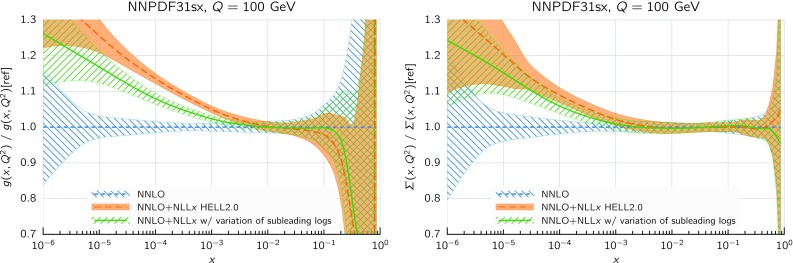
Comparison between the gluon (left) and the total quark singlet (right plots) from the NNLO and $\hbox {NNLO+NLL}x$ DIS-only fits, including the variant of the resummation which differs by subleading terms, as discussed in the text

In Fig. 8 we observed that including resummation leads to a significantly larger shift in the small-*x* quark singlet and gluon PDFs at NNLO than at NLO. This is so despite the fact that from the point of view of small-*x* resummation the information added is the same in both cases, and that the resummed splitting and coefficient functions at small *x* are quite similar whichever fixed-order calculation they are matched to. The explanation of this paradoxical result is that fixed-order perturbation theory is unstable at small *x* due to the small-*x* logarithms, and while this instability is quite small at NLO, due to accidental zeros in some of the coefficients, it is significant at NNLO, and would probably become very substantial at $\hbox {N}^3\hbox {LO}$. To better illustrate this effect, and the way it is cured by resummation, in Fig. 9 we compare the NLO, NNLO and $\hbox {NNLO+NLL}x$ results for the gluon and singlet PDFs in the baseline fits at $Q=100$ GeV, normalized to the NLO prediction. We find that the NNLO results are systematically below the NLO ones for $x\le 10^{-2}$, and that the net effect of adding NLL*x* resummation to the NNLO fit is to bring it more in line with the NLO (and thus as well with the $\hbox {NLO+NLL}x$) result. This provides an explanation of our previous observation that NNLO theory fits small-*x* DIS data worse than NLO, while $\hbox {NNLO+NLL}x$ provides the best description of all.

So far we focused on the gluon and quark singlet, as small-*x* resummation affects PDFs in the singlet sector. To quantify the effect of resummation on the PDFs in the physical basis it is convenient to use a distance estimator, as defined in Refs. [74, 77]. This allows us to represent in a concise way how two PDF fits differ among themselves, both at the level of central values and of PDF uncertainties. In Fig. 10 we show these distances between the central values (left) and the PDF uncertainties (right) of the NNPDF3.1sx NNLO and $\hbox {NNLO+NLL}x$ fits at $Q=100$ GeV. Since these fits are based on $N_{\mathrm{rep}}=100$ replicas each, a distance of $d\sim 10$ corresponds to a variation of one sigma of the central values or the PDF uncertainties in units of the corresponding standard deviation.

From the comparison in Fig. 10 we see that the impact of using $\hbox {NNLO+NLL}x$ theory peaks between $x\simeq 10^{-3}$ and $x\simeq 10^{-5}$, where $d\gtrsim 30$, meaning that the central value shifts by more than three times the corresponding PDF uncertainty. The gluon is the most affected PDF, followed by the charm and then by the light quark PDFs. Note that the differences are not restricted to the region of very small-*x*, since for gluons $d\sim 10$ already at $x\simeq 5\cdot 10^{-3}$, relevant for the production of electroweak scale particles such as *W* and *Z* bosons at the LHC. On the other hand, the impact of using $\hbox {NNLO+NLL}x$ theory is as expected small for the PDF uncertainties, since from the experimental point of view very little new information is being added into the fit. However, as we will discuss in greater detail in Sect. 4.2.4, adding small-*x* resummation has allowed us to lower the minimum value of $Q^2$ for the HERA data included in the fits – which in turn extends to smaller *x* the PDF kinematic coverage, thus reducing PDF uncertainties in the very small-*x* region.

Before moving to the global fits, we want to briefly investigate how our results are sensitive to unknown subleading logarithmic contributions. Indeed, the results of Ref. [63] are provided with an uncertainty band aimed at estimating the impact of subleading (NNLL*x*) contributions not predicted by NLL*x* resummation. Ideally, the uncertainty band should be included as a theory uncertainty in the fit procedure; however, at the moment the inclusion of theory uncertainties in PDF fits is still under study. Nevertheless, we can investigate the effects of such uncertainties by performing another fit in which we change the resummation by subleading terms. A simple way to do it in a consistent manner is to vary by subleading terms the anomalous dimension used for the resummation of coefficient functions and of $P_{qg}$. As the resummed gluon splitting function depends on the resummed $P_{qg}$, all splitting functions and coefficient functions are affected by this change. More specifically, the so-called LL$^\prime$ anomalous dimension used in HELL 2.0 (and hence in this work) is replaced with the full NLL*x* anomalous dimension, as proposed originally in Ref. [46]. The effect of this variation is contained within the uncertainty bands of Ref. [63].

The result of this fit, based on the same DIS-only dataset considered so far and performed at $\hbox {NNLO+NLL}x$ accuracy, is fully consistent with that obtained with the baseline theory settings. The fit quality is essentially unaffected, and the $\chi ^2$ variations with respect to the numbers in Table 3 are compatible with statistical fluctuations. Most PDFs are not sensitive to this variation, except the gluon and the quark singlet, which do change a little, to accommodate the different subleading terms in the splitting functions and coefficient functions. These PDFs are shown in Fig. 11 and compared with the default HELL 2.0 result. In both cases the new PDFs are smaller than our default ones, i.e. closer to the NNLO results. This is mostly due to a harder resummed $P_{qg}$ in the varied resummation, which is therefore closer to its NNLO counterpart, at intermediate values of *x*, than our default resummation. For the gluon in particular, the new results are not compatible within the uncertainty bands with our default fit, highlighting that the PDF uncertainty does not cover the theory uncertainty from missing higher orders. However, all the qualitative conclusions remain unchanged.

### Global fits

We now turn to consider the global fits, based on the complete dataset described in Sect. 3.2. We first show the results of the fits, obtained with the default cut parameter $H_{\mathrm{cut}}=0.6$, highlighting similarities and differences with respect to the DIS-only fits, and we discuss the impact of resummation on the PDFs. We then study the dependence of our results upon variation of the value of $H_{\mathrm{cut}}$. Finally, we discuss in some detail the description of the low-$Q^2$ HERA bin which we include in the NNPDF31sx fits.

#### Fit results and comparison to the DIS-only fits

**Table 4:** Same as Table 3, now for the global NNPDF3.1sx NLO, $\hbox {NLO+NLL}x$, NNLO and $\hbox {NNLO+NLL}x$ fits, corresponding to the baseline value of $H_{\mathrm{cut}}=0.6$ for the cut to the hadronic data

	$\chi ^2/N_{\mathrm{dat}}$		$\Delta \chi ^2$	$\chi ^2/N_{\mathrm{dat}}$		$\Delta \chi ^2$
	NLO	$\hbox {NLO+NLL}x$		NNLO	$\hbox {NNLO+NLL}x$	
NMC	1.35	1.35	$+1$	1.30	1.33	$+9$
SLAC	1.16	1.14	−1	0.92	0.95	$+2$
BCDMS	1.13	1.15	$+12$	1.18	1.18	$+3$
CHORUS	1.07	1.10	$+20$	1.07	1.07	−2
NuTeV dimuon	0.90	0.84	−5	0.97	0.88	−7
HERA I+II incl. NC	1.12	1.12	$-2$	1.17	1.11	$-62$
HERA I+II incl. CC	1.24	1.24	–	1.25	1.24	$-1$
HERA $\sigma _c^{\mathrm{NC}}$	1.21	1.19	−1	2.33	1.14	−56
HERA $F_2^b$	1.07	1.16	$+3$	1.11	1.17	$+2$
DY E866 $\sigma ^d_{\mathrm{DY}}/\sigma ^p_{\mathrm{DY}}$	0.37	0.37	–	0.32	0.30	–
DY E886 $\sigma ^p$	1.06	1.10	$+3$	1.31	1.32	–
DY E605 $\sigma ^p$	0.89	0.92	$+3$	1.10	1.10	–
CDF *Z* rap	1.28	1.30	–	1.24	1.23	–
CDF Run II $k_t$ jets	0.89	0.87	$-2$	0.85	0.80	−4
D0 *Z* rap	0.54	0.53	–	0.54	0.53	–
D0 $W\rightarrow e\nu$ asy	1.45	1.47	–	3.00	3.10	$+1$
D0 $W\rightarrow \mu \nu$ asy	1.46	1.42	–	1.59	1.56	–
ATLAS total	1.18	1.16	$-7$	0.99	0.98	$-2$
ATLAS *W*, *Z* 7 TeV 2010	1.52	1.47	–	1.36	1.21	$-1$
ATLAS HM DY 7 TeV	2.02	1.99	–	1.70	1.70	–
ATLAS *W*, *Z* 7 TeV 2011	3.80	3.73	$-1$	1.43	1.29	$-1$
ATLAS jets 2010 7 TeV	0.92	0.87	$-4$	0.86	0.83	$-2$
ATLAS jets 2.76 TeV	1.07	0.96	$-6$	0.96	0.96	–
ATLAS jets 2011 7 TeV	1.17	1.18	–	1.10	1.09	$-1$
ATLAS *Z* $p_T$ 8 TeV $(p_T^{ll},M_{ll})$	1.21	1.24	+2	0.94	0.98	$+2$
ATLAS *Z* $p_T$ 8 TeV $(p_T^{ll},y_{ll})$	3.89	4.26	+2	0.79	1.07	$+2$
ATLAS $\sigma _{tt}^{tot}$	2.11	2.79	+2	0.85	1.15	$+1$
ATLAS $t\bar{t}$ rap	1.48	1.49	–	1.61	1.64	–
CMS total	0.97	0.92	$-13$	0.86	0.85	$-3$
CMS Drell–Yan 2D 2011	0.77	0.77	–	0.58	0.57	–
CMS jets 7 TeV 2011	0.88	0.82	$-9$	0.84	0.81	$-3$
CMS jets 2.76 TeV	1.07	0.98	$-7$	1.00	1.00	–
CMS *Z* $p_T$ 8 TeV $(p_T^{ll},y_{ll})$	1.49	1.57	$+1$	0.73	0.77	–
CMS $\sigma _{tt}^{tot}$	0.74	1.28	$+2$	0.23	0.24	–
CMS $t\bar{t}$ rap	1.16	1.19	–	1.08	1.10	–
Total	1.117	1.120	$+11$	1.130	1.100	$-121$

We start by considering the quality of the global NNPDF3.1sx fits at NLO, $\hbox {NLO+NLL}x$, NNLO and $\hbox {NNLO+NLL}x$, using the default value of $H_{\mathrm{cut}}=0.6$ for the hadronic data cut discussed in Sect. 3.2. The values of the $\chi ^2/N_{\mathrm{dat}}$ for the total and the individual datasets are shown in Table 4. As in the DIS-only case, in this table we also include the absolute $\chi ^2$ difference between the resummed and fixed-order results, $\Delta \chi ^2$ Eq. (4.1). We observe that the NNPDF3.1sx fit based on $\hbox {NNLO+NLL}x$ theory leads to the best overall fit quality, $\chi ^2/N_{\mathrm{dat}}= 1.100$. The NNLO fit, on the other hand, has again the highest $\chi ^2/N_{\mathrm{dat}}=1.130$, so that the overall improvement is $\Delta \chi ^2=-121$. Whilst resummation proves particularly beneficial at NNLO, the effect at NLO is very mild; the $\chi ^2/N_{\mathrm{dat}}\simeq 1.120$ at $\hbox {NLO+NLL}x$ is compatible, within statistical fluctuations, with the 1.117 obtained with fixed-order theory, that is, $\Delta \chi ^2=+11$. Note that in the NNPDF3.1 fits the NNLO $\chi ^2$ was markedly better than the NLO one [79]: this is no longer the case here, since the high-precision Drell–Yan and *Z* $p_T$ data points, which are poorly described by NLO theory, are now partly removed by the $H_{\mathrm{cut}}$ cut.

The improvement of the $\chi ^2$ at $\hbox {NNLO+NLL}x$ is essentially due to the HERA charm and neutral-current structure function data. On one hand, as we already noticed in the DIS-only fits, by using $\hbox {NNLO+NLL}x$ theory one achieves an improved description of the precise HERA NC inclusive structure function measurements, whose $\chi ^2/N_{\mathrm{dat}}$ decreases from 1.17 in the NNLO fit to 1.11 in the $\hbox {NNLO+NLL}x$ fit, $\Delta \chi ^2=-62$. A marked improvement is also achieved for the HERA charm cross-sections, whose $\chi ^2/N_{\mathrm{dat}}$ goes down from 2.33 to 1.14, $\Delta \chi ^2=-56$. These two datasets are thus sufficient to explain the overall improvement in the total $\chi ^2$.

We also find that NNLO theory describes better than the corresponding NLO theory the ATLAS and CMS measurements, particularly the recent high-precision data such as the ATLAS *W*, *Z* 2011 rapidity distributions, and the ATLAS and CMS 8 TeV *Z* $p_T$ distributions. Specifically, the $\chi ^2/N_{\mathrm{dat}}$ total values for ATLAS and CMS is $1.18\, (1.16)$ and $0.97\, (0.92)$ in the NLO(+NLL*x*) fits, respectively, decreasing to $0.99\, (0.98)$ and $0.86\, (0.85)$ when using NNLO (+NLL*x*) theory. It is interesting that in all cases the resummed fits are slightly better than their fixed-order counterparts.

Despite the improved description of the large-$Q^2$ collider data with respect to the NLO theory, the NNLO fit turns out to have the highest $\chi ^2$ of the four theories, as in the DIS-only case. The main reason is the poor description of the HERA inclusive and charm dataset, which contain almost one third ($N_{\mathrm{dat}}=1209$) of the number of data points included in the fit ($N_{\mathrm{dat}}=3930$). Moreover, we observe that the effects of small-*x* resummation at NNLO are confined to the HERA data; the differences between the $\chi ^2$ values of the (N)NLO and (N)$\hbox {NLO+NLL}x$ fits for the other datasets are being all rather small. This is in agreement with the findings of the DIS-only fits, and with the fact that hadronic data potentially sensitive to small-*x* effects have been cut. Specifically, in the NNLO fits there is no other dataset besides the HERA inclusive and charm data with $|\Delta \chi ^2|\ge 10$.

Comparing the values of the $\chi ^2/N_{\mathrm{dat}}$ for the DIS experiments in the global and DIS-only fits, we notice that once resummation is accounted for, the global fit is if anything slightly better than the DIS-only fit. In particular for the inclusive HERA data, where $\chi ^2/N_{\mathrm{dat}}$ is 1.16 (1.12) at NNLO(+NLL*x*) in the DIS-only fits, we have $\chi ^2/N_{\mathrm{dat}} = 1.17~(1.11)$ in the global fits, so that $\Delta \chi ^2$ decreases from $-47$ to $-62$ in the global fit. The other significant difference between the global and DIS-only fits appears in the NuTeV dimuon data, which is fit somewhat less well in the global fit (irrespective of resummation) due to the tension with the LHC data relative to the proton strangeness, especially with the ATLAS *W*, *Z* 2011 rapidity distributions [79].

**Fig. 12:**
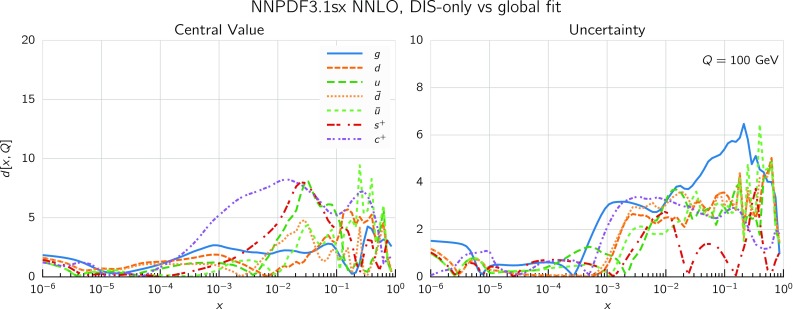
Same as Fig. 10 for the comparison between the fixed-order NNLO NNPDF3.1sx DIS-only and global fits. Note that the range of the *y* axis on the left plot has been reduced

We now move to the impact of small-*x* resummation on the global dataset PDFs. First, we quantify the differences between the global and the DIS-only fits, taking as a representative the baseline fixed-order NNLO fit. We start by showing the distance estimator in Fig. 12, both for the central value (left) and the PDF uncertainty (right), at $Q=100$ GeV. Due to the conservative kinematic cut imposed on the collider observables, the distances between the global and DIS-only fits are moderate and localized to the medium and large-*x* region, while the small-*x* region is pretty much unchanged. The PDF flavor which is most affected is the charm PDF, whose distance is about 10 for $x\sim 10^{-2}$. The decrease in PDF uncertainties in the global dataset at medium and large-*x* is clearly visible, especially for the gluon PDF which is only constrained in an indirect way by the DIS structure function data.

**Fig. 13:**
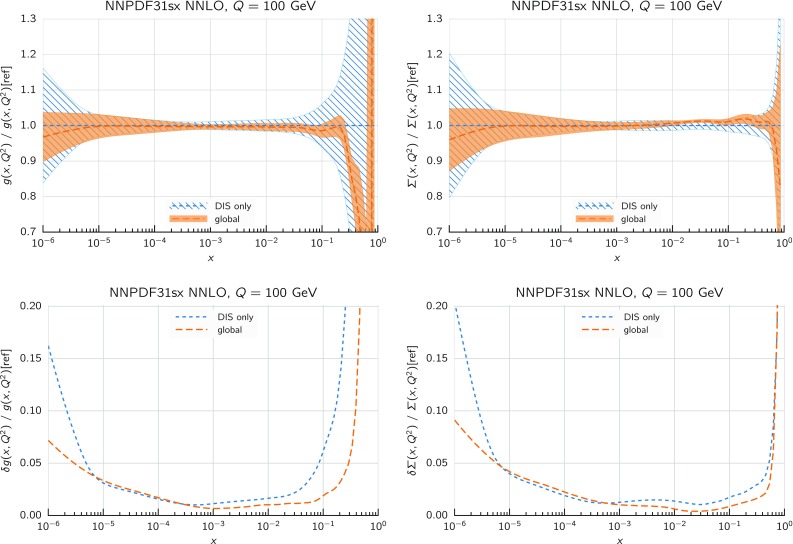
Comparison between the gluon (left) and the total quark singlet (right) at $Q=100$ GeV between the NNPDF3.1sx NNLO DIS-only and global fits. The upper plots show the ratio of global fit results over the DIS-only fit results, while the bottom plots compare the relative PDF uncertainty between the two fits

**Fig. 14:**
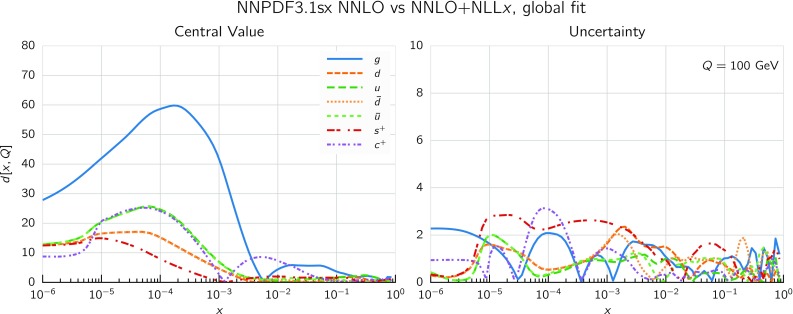
Same as Fig. 10 for the NNPDF3.1sx global fits

In Fig. 13 we show a direct comparison between the gluon (left) and the total quark singlet (right) at $Q=100$ GeV between the NNPDF3.1sx NNLO DIS-only and global fits. The upper plots show the ratio of global fit results over the DIS-only fit results, while the bottom plots compare the relative PDF uncertainty between the two fits. At the level of central values, there is good consistency at the one-sigma level; for $x\lesssim 0.1$, the central values of the DIS-only and global fits are very close to each other. Concerning PDF uncertainties, the improvement in going from DIS-only to global is very clear, especially in the large-*x* region for the gluon where the DIS-only fit exhibits much larger uncertainties. The global fit also exhibits somewhat smaller uncertainties in the extrapolation region for $x \lesssim 10^{-5}$, even if at small *x* the direct constraints are essentially the same in the two cases. However, given the large size of PDF uncertainties in this region, the observed differences are consistent with statistical fluctuations.

#### Features of the small-*x* resummed PDFs from the global fit

The comparison done so far demonstrates that the use of the global dataset is very beneficial from the point of view of the PDF uncertainties, while it does not affect the qualitative and quantitative results at small *x*. Therefore, the global fits will be considered from now on the baseline NNPDF3.1sx fits, and we will focus on these results for subsequent applications and studies. Therefore, before moving forward, it is interesting to analyse the features of these fits in more detail.

We focus on the results at NNLO and $\hbox {NNLO+NLL}x$, as at NLO the impact of resummation is less significant (just as in the DIS-only fits) and also less important from the point of view of applications to the LHC and future high-energy collider physics. In Fig. 14 we show the same distance comparison as in Fig. 10 but now for the NNPDF3.1sx global fits. By comparing this figure with the corresponding DIS-only case, we see that in the global fits the qualitative features are the same. The increased significance of the distances at large *x* observed in the global fit as compared to the DIS-only is a direct consequence of the reduced PDF uncertainties in the global fit, rather than to a shift in the central values.

**Fig. 15:**
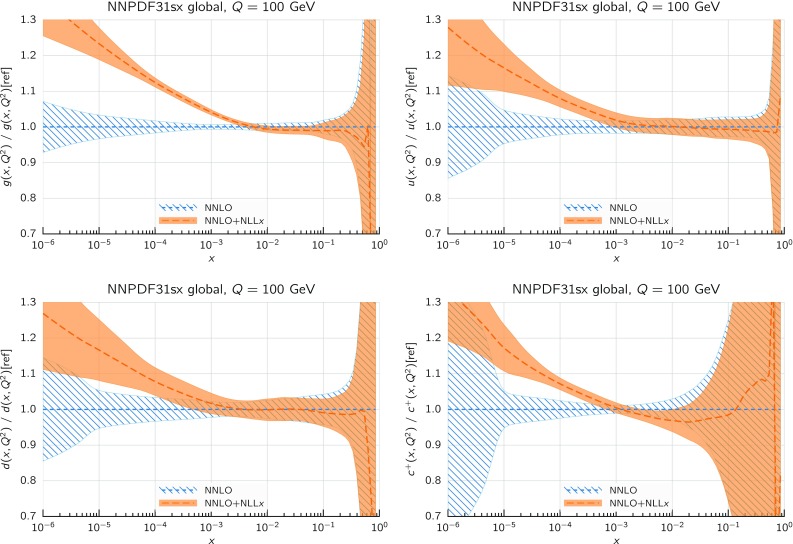
Comparison of the NNPDF3.1sx NNLO and $\hbox {NNLO+NLL}x$ global fits at $Q=100$ GeV. We show the gluon PDF and the charm, up, and down quark PDFs, normalized to the central value of the baseline NNLO fit

**Fig. 16:**
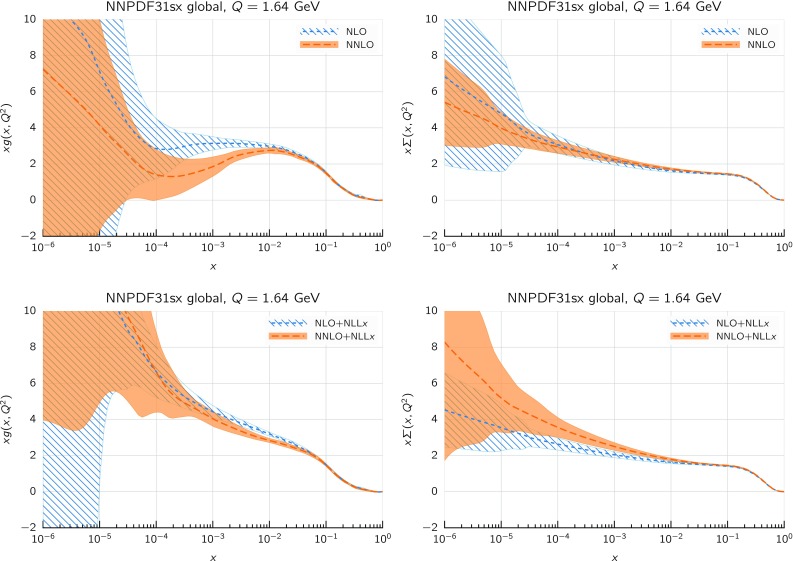
Comparison of NLO and NNLO fit results at the input parametrization scale of $Q=1.64$ GeV (upper plots), and of $\hbox {NLO+NLL}x$ and $\hbox {NNLO+NLL}x$ fit results at the input parametrization scale of $Q=1.64$ GeV (lower plots). Left plots: gluon; right plots: quark singlet

To visualize these effects, in Fig. 15 we show the flavor combinations most affected by resummation (as indicated in the distance plot of Fig. 14), namely the gluon, charm, up and down PDFs, at a typical electroweak scale of $Q=100$ GeV. The impact of NLL*x* resummation is very similar for all the quark combinations: the effect is mild for $x\gtrsim 10^{-3}$, whilst it increases at small *x*, by an amount which is, however, mostly consistent with the one or two sigma PDF uncertainties. The effect is rather more marked for the gluon, where the $\hbox {NNLO+NLL}x$ fit can be up to 30% bigger at $x\simeq 10^{-6}$, well outside the uncertainty band. Thus the main impact of high-energy resummation is to strongly enhance the gluon and mildly enhance the quarks at small-*x*.

To conclude the discussion of the results of the global NNPDF3.1sx fits, we move away from the electroweak scale and consider the PDFs at the input parametrization scale $Q_0$. This comparison is interesting because it disentangles the effects of small-*x* resummation on the fitted PDFs from those due to the evolution from low to high scales. With this motivation, we show in Fig. 16 the gluon and the quark singlet at the fit scale $Q_0=1.64$ GeV. In the case of the total quark singlet, we see that the impact of resummation is moderate, with a one-sigma increase at small *x* in the $\hbox {NNLO+NLL}x$ fit which helps to improve the fit to the low $Q^2$ HERA data. The slightly larger effects seen at higher scales are thus mostly driven by the evolution that mixes the singlet with the gluon. On the other hand, the effects of resummation are more marked for the fitted gluon, where we see explicitly a drop in the NNLO gluon at small *x* driven by perturbative instability, which disappears on resummation in such a way that the $\hbox {NNLO+NLL}x$ gluon is rather flat, and indeed very close to the NLO and $\hbox {NLO+NLL}x$ gluon. Note that the resummation thus extends the perturbative region at small *x*: even at $Q_0=1.64$ GeV the fitted gluon remains stable, and it seems likely that one would have to go to even lower scales (below the charm threshold) before the kind of instability seen in NNLO fixed-order perturbation theory sets in. Note that we would not expect the same to be true of $\hbox {N}^3\hbox {LO}$ perturbation theory: the unresummed logarithms at $\hbox {N}^3\hbox {LO}$ are considerably larger than those at NNLO, and thus the need for resummation at $\hbox {N}^3\hbox {LO}$ would be even more pressing than at NNLO.

#### Dependence on the value of $H_{\mathrm{cut}}$

Thus far we have only discussed the results of the global fit obtained using the default cut to the hadronic data, identified as $H_{\mathrm{cut}}=0.6$. We now discuss the dependence of the fit results with respect to variations of this choice, both from the point of view of the fit quality and of the impact at the PDF level. In doing so, we provide further motivation for the choice of $H_{\mathrm{cut}}=0.6$ for our default global fits.

To begin with, we study the dependence of the quality of the NNPDF3.1sx fits as a function of the value of the cut parameter $H_{\mathrm{cut}}$ applied to the hadronic data. In Table 5 we show a comparison of the NNLO and $\hbox {NNLO+NLL}x$ values of the $\chi ^2/N_{\mathrm{dat}}$ for the fits with $H_{\mathrm{cut}}=0.5,0.6$ and 0.7. In addition, to better appreciate the variations for $\chi ^2$ for the fits with different $H_{\mathrm{cut}}$ cuts, in Table 6 we also show the differences

4.2 $$\begin{aligned} \delta \chi ^2\equiv \chi ^2 \left( H_{\mathrm{cut}}^{(1)}\right) -\chi ^2\left( H_{\mathrm{cut}}^{(2)}\right) , \end{aligned}$$

for the global fits obtained using NNLO and $\hbox {NNLO+NLL}x$ theory. To highlight that in general fits varying $H_{\mathrm{cut}}$ have different number of data points, we also indicate in the same table the difference $\delta N_{\mathrm{dat}}=N_{\mathrm{dat}}\left( H_{\mathrm{cut}}^{(1)}\right) -N_{\mathrm{dat}}\left( H_{\mathrm{cut}}^{(2)}\right)$ for each experiment.

The main general feature that we note from the comparisons in Tables 5 and 6 is that the $\chi ^2/N_{\mathrm{dat}}$ values exhibit a rather moderate dependence on the specific value of the kinematic cut to the hadronic data.

Concerning the total dataset, the $\chi ^2/N_{\mathrm{dat}}$ values slightly increase as $H_{\mathrm{cut}}$ is raised and the dataset is enlarged: in particular, for the NNLO ($\hbox {NNLO+NLL}x$) fits, the values of $\chi ^2/N_{\mathrm{dat}}$ for the total dataset are 1.120, 1.130, and 1.142 (1.085, 1.100, and 1.112) for $H_{\mathrm{cut}}=0.5, 0.6$ and 0.7 respectively. The fact that the fit quality of both the fixed-order and the resummed fits is slightly better for $H_{\mathrm{cut}}=0.5$ is a direct consequence of the more restrictive dataset.

In the case of the $\hbox {NNLO+NLL}x$ fits, the difference between the $\chi ^2/N_{\mathrm{dat}}$ of the fit with $H_{\mathrm{cut}}=0.6$ and the fit with $H_{\mathrm{cut}}=0.7$ is larger than a statistical fluctuation. This might be an indication that the deterioration of the fit with $H_{\mathrm{cut}}=0.7$ could be related to non-negligible effects of unresummed small-*x* logarithms in the extra hadronic data that are included in this fit. This conjecture is supported by the fact that, while with $H_{\mathrm{cut}}=0.6$ the resummation improves the total $\chi ^2$ over the fixed order by around 120 points, for $H_{\mathrm{cut}}=0.7$ the improvement is reduced to less than 100 points. On the other hand, the same trend is also visible in the NNLO fits, and there it can be partly explained by the contributions from some collider points that are in tension between the DIS data, for instance, the ATLAS *W*, *Z* 2011 rapidity distributions and the neutrino data. We also find that the more conservative fit with $H_{\mathrm{cut}}=0.5$ also improves with resummation by even more than the $H_{\mathrm{cut}}=0.6$ fit (around 140 points), thus suggesting that our default cut value is safe, in the sense that it is not affected by large unresummed logarithms in the hadronic processes.

**Table 5:** Same as Table 4, now comparing the values of the $\chi ^2/N_{\mathrm{dat}}$ for the global NNLO and $\hbox {NNLO+NLL}x$ fits obtained with different values of the hadronic data cut, $H_{\mathrm{cut}}=0.5, 0.6$ and 0.7. Note that fits with different values of $H_{\mathrm{cut}}$ have in general a different number of data points in the hadronic experiments, as indicated in Table 2. Columns 4 and 5 of this table correspond to the same numbers as those in columns 5 and 6 of Table 4. For ease of comparison, the $\delta \chi ^2$ variations among fits with different cuts, Eq. (4.2), are collected in Table 6

	$H_{\mathrm{cut}}=0.5$		$H_{\mathrm{cut}}=0.6$		$H_{\mathrm{cut}}=0.7$	
	NNLO	$\hbox {NNLO+NLL}x$	NNLO	$\hbox {NNLO+NLL}x$	NNLO	$\hbox {NNLO+NLL}x$
NMC	1.31	1.31	1.30	1.33	1.31	1.36
SLAC	1.03	0.96	0.92	0.95	0.92	0.88
BCDMS	1.18	1.18	1.18	1.18	1.18	1.14
CHORUS	1.04	1.03	1.07	1.07	1.10	1.10
NuTeV dimuon	0.68	0.82	0.97	0.88	0.91	1.06
HERA I+II incl. NC	1.17	1.11	1.17	1.11	1.17	1.12
HERA I+II incl. CC	1.23	1.23	1.23	1.24	1.26	1.26
HERA $\sigma _c^{\mathrm{NC}}$	2.34	1.17	2.33	1.14	2.43	1.17
HERA $F_2^b$	1.10	1.16	1.11	1.17	1.11	1.17
DY E866 $\sigma ^d_{\mathrm{DY}}/\sigma ^p_{\mathrm{DY}}$	0.34	0.35	0.32	0.30	0.38	0.36
DY E886 $\sigma ^p$	0.99	0.96	1.31	1.32	1.33	1.28
DY E605 $\sigma ^p$	1.05	1.03	1.10	1.10	1.17	1.10
CDF *Z* rap	1.49	1.47	1.24	1.23	1.55	1.46
CDF Run II $k_t$ jets	0.83	0.80	0.85	0.80	0.85	0.86
D0 *Z* rap	0.71	0.72	0.54	0.53	0.65	0.64
D0 $W\rightarrow e\nu$ asy	4.16	4.18	3.00	3.10	2.85	2.90
D0 $W\rightarrow \mu \nu$ asy	1.78	1.81	1.59	1.56	1.41	1.50
ATLAS total	1.00	0.97	0.99	0.98	1.05	1.01
ATLAS *W*, *Z* 7 TeV 2010	–	–	1.36	1.21	1.07	0.95
ATLAS HM DY 7 TeV	1.55	1.61	1.70	1.70	1.62	1.72
ATLAS *W*, *Z* 7 TeV 2011	–	–	1.43	1.29	2.11	1.75
ATLAS jets 2010 7 TeV	0.88	0.82	0.86	0.83	0.92	0.89
ATLAS jets 2.76 TeV	0.94	0.87	0.96	0.96	0.98	0.93
ATLAS jets 2011 7 TeV	1.09	1.08	1.10	1.09	1.11	1.08
ATLAS *Z* $p_T$ 8 TeV $(y_{ll},M_{ll})$	0.99	1.04	0.94	0.98	0.94	0.98
ATLAS *Z* $p_T$ 8 TeV $(p_T^{ll},M_{ll})$	–	–	0.79	1.07	0.61	0.73
ATLAS $\sigma _{tt}^{tot}$	0.91	1.22	0.85	1.15	0.84	1.12
ATLAS $t\bar{t}$ rap	1.76	1.73	1.61	1.64	1.55	1.56
CMS total	0.88	0.84	0.86	0.85	0.90	0.88
CMS *W* asy 840 pb	–	–	–	–	0.41	0.39
CMS *W* asy 4.7 fb	–	–	–	–	1.25	1.23
CMS Drell–Yan 2D 2011	0.57	0.84	0.58	0.51	0.95	1.01
CMS *W* rap 8 TeV	–	–	–	–	0.85	0.64
CMS jets 7 TeV 2011	0.83	0.76	0.84	0.81	0.84	0.81
CMS jets 2.76 TeV	1.00	0.95	1.00	1.00	1.00	0.98
CMS *Z* $p_T$ 8 TeV $(p_T^{ll},M_{ll})$	1.20	1.55	0.73	0.77	0.74	0.77
CMS $\sigma _{tt}^{tot}$	0.24	0.28	0.23	0.24	0.23	0.23
CMS $t\bar{t}$ rap	0.78	0.78	1.08	1.10	0.91	0.92
Total	1.120	1.085	1.130	1.100	1.142	1.112

**Table 6:** The differences $\delta \chi ^2\equiv \chi ^2\left( H_{\mathrm{cut}}^{(1)}\right) -\chi ^2\left( H_{\mathrm{cut}}^{(2)}\right)$, Eq. (4.2), for the global fits reported in Table 5. Since the fits with different values of $H_{\mathrm{cut}}$ have in general a different number of data points for the hadronic experiments, we also indicate in each case the difference $\delta N_{\mathrm{dat}}=N_{\mathrm{dat}}\left( H_{\mathrm{cut}}^{(1)}\right) -N_{\mathrm{dat}}\left( H_{\mathrm{cut}}^{(2)}\right)$

	$\chi ^2(0.6)-\chi ^2(0.5)$		$\delta N_{\mathrm{dat}}$	$\chi ^2(0.7)-\chi ^2(0.6)$		$\delta N_{\mathrm{dat}}$
	NNLO	$\hbox {NNLO+NLL}x$		NNLO	$\hbox {NNLO+NLL}x$	
NMC	$-4$	$+6$	–	$+2$	$+12$	–
SLAC	$-9$	$-1$	–	–	$-5$	–
BCDMS	$-2$	$-1$	–	–	$-21$	–
CHORUS	$+32$	$+38$	–	$+20$	$+28$	–
NuTeV dimuon	$+23$	$+5$	–	$-5$	$+14$	–
HERA I+II incl NC	$-5$	$-7$	–	$+2$	$+11$	–
HERA I+II incl CC	$+2$	$+1$	–	$+1$	$+2$	–
HERA $\sigma _c^{\mathrm{NC}}$	$-1$	$-1$	–	$+4$	$+1$	–
HERA $F_2^b$	–	–	–	–	$+1$	–
DY E866 $\sigma ^d_{\mathrm{DY}}/\sigma ^p_{\mathrm{DY}}$	–	–	$+2$	–	$+1$	$+1$
DY E886 $\sigma ^p$	$+44$	$+47$	$+20$	$+17$	$+12$	$+12$
DY E605 $\sigma ^p$	$+5$	$+7$	–	$+5$	–	–
CDF *Z* rap	$+7$	$+7$	$+8$	$+20$	$+17$	$+9$
CDF Run II $k_t$ jets	$+1$	–	–	–	$+5$	–
D0 *Z* rap	$+2$	$+2$	$+8$	$+8$	$+7$	$+8$
D0 $W\rightarrow e\nu$ asy	$+4$	$+5$	$+3$	$+2$	$+1$	$+1$
D0 $W\rightarrow \mu \nu$ asy	$+6$	$+5$	$+4$	–	$+1$	$+1$
ATLAS total	$+25$	$+29$	$+27$	$+53$	$+43$	$+36$
ATLAS *W*, *Z* 7 TeV 2010	$+8$	$+7$	$+6$	$+8$	$+8$	$+10$
ATLAS HM DY 7 TeV	$+1$	–	–	–	–	$+1$
ATLAS *W*, *Z* 7 TeV 2011	$+11$	$+10$	$+8$	$+30$	$+25$	$+12$
ATLAS jets 2010 7 TeV	$+3$	$+5$	$+5$	$+7$	$+7$	$+3$
ATLAS jets 2.76 TeV	$+4$	$+7$	$+3$	$+1$	$-2$	–
ATLAS jets 2011 7 TeV	–	–	–	–	–	–
ATLAS *Z* $$p_T$$ 8 TeV $$(y_{ll},M_{ll}) $$	$$+5$$	$$+6$$	$$+6$$	$$+6$$	$$+6$$	$$+11$$
ATLAS *Z* $p_T$ 8 TeV $(p_T^{ll},M_{ll})$	$-2$	$-3$	–	–	–	–
ATLAS $\sigma _{tt}^{tot}$	–	–	–	–	–	–
ATLAS $t\bar{t}$ rap	$-2$	$-1$	–	$-1$	$-1$	–
CMS total	$+17$	$+24$	$+25$	$+60$	$+57$	$+57$
CMS *W* asy 840 pb	–	–	–	$+8$	$+8$	$+7$
CMS *W* asy 4.7 fb	–	–	–	$+10$	$+9$	$+7$
CMS Drell–Yan 2D 2011	$+9$	$+7$	–	$+28$	$+31$	$+20$
CMS *W* rap 8 TeV	–	–	$+16$	$+11$	$+11$	$+12$
CMS jets 7 TeV 2011	$+1$	$+7$	–	–	–	–
CMS jets 2.76 TeV	–	$+4$	–	–	$-2$	–
CMS *Z* $p_T$ 8 TeV $(p_T^{ll},M_{ll})$	$+4$	$+3$	$+7$	$+6$	$+7$	$+9$
CMS $\sigma _{tt}^{tot}$	–	–	–	–	–	–
CMS $t\bar{t}$ rap	$+4$	$+4$	$+2$	–	–	$+2$

**Fig. 17:**
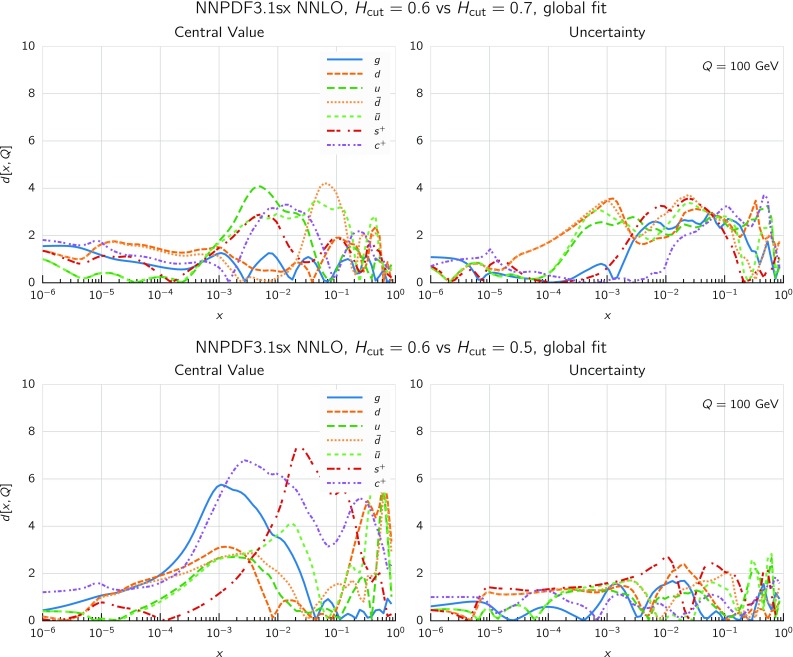
Same as Fig. 10 for the comparison of the baseline NNPDF3.1sx NNLO+NLL global fit with $H_{\mathrm{cut}}=0.6$ with the corresponding fits with $H_{\mathrm{cut}}=0.7$ (upper) and $H_{\mathrm{cut}}=0.5$ (lower plots)

We further investigate the impact on the PDFs of the various choices of $H_{\mathrm{cut}}$. We show in Fig. 17 the distance estimator to compare the default NNPDF3.1sx $\hbox {NNLO+NLL}x$ global fit with $H_{\mathrm{cut}}=0.6$ with the corresponding fits with the $H_{\mathrm{cut}}=0.7$ and $H_{\mathrm{cut}}=0.5$ fits. In terms of central values, we see that differences are well below PDF uncertainties (which corresponds to $d\simeq 10$) when comparing $H_{\mathrm{cut}}=0.7$ to $H_{\mathrm{cut}}=0.6$. On the contrary, the distances between the $H_{\mathrm{cut}}=0.5$ fit and the $H_{\mathrm{cut}}=0.6$ fit are larger, especially for charm and strangeness at $x \gtrsim 10^{-3}$. This comparison indicates that there is no real benefit in loosening the cut from $H_{\mathrm{cut}}=0.6$ to $H_{\mathrm{cut}}=0.7$ (since differences at the PDF level are small, and the possibility of biasing the fit higher) whilst it is indeed advantageous to use $H_{\mathrm{cut}}=0.6$ rather than the tighter cut $H_{\mathrm{cut}}=0.5$, thanks to the increase in PDF constraints provided by the additional data.

**Fig. 18:**
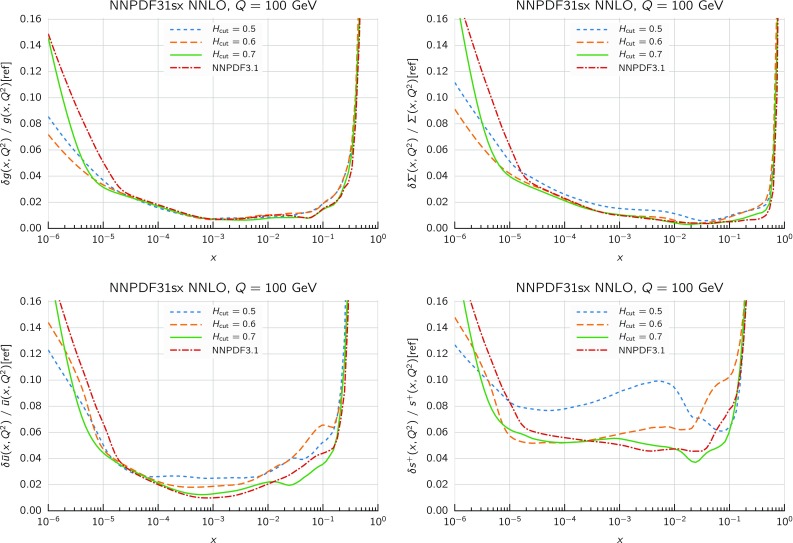
The relative PDF uncertainties in the NNPDF3.1sx NNLO fits with the three different values of the $H_{\mathrm{cut}}$ cut on the hadronic data, compared with those from NNPDF3.1. We show the gluon, the quark singlet, the anti-up quark, and the total strangeness, at $Q=100$ GeV

Finally, in Fig. 18 we show the relative PDF uncertainties in the NNPDF3.1sx NNLO fits with the three different values of the $H_{\mathrm{cut}}$ cut on the hadronic data. For completeness, we also include in this comparison the results of the NNPDF3.1 fit. Specifically, we show the gluon, the quark singlet, the anti-up quark, and the total strangeness, at $Q=100$ GeV. From the comparison we see that, as expected, the smaller the value of $H_{\mathrm{cut}}$, the more marked the increase in PDF uncertainties. On the other hand, we see that for $H_{\mathrm{cut}}=0.6$ the results are already competitive with those of NNPDF3.1. We also find that in the small-*x* region, PDF uncertainties are smaller in the NNPDF3.1sx fits than in the NNPDF3.1 ones, especially for our default value of $H_{\mathrm{cut}}=0.6$, due to the lowering of the $Q_{\mathrm{min}}^2$ kinematic cut (see also the discussion in Sect. 4.2.4).

Summarizing, we have provided here a number of indications that the NNPDF3.1sx fit with $H_{\mathrm{cut}}=0.6$ is not biased by hadronic data sensitive to small-*x* resummation, and at the same time we have demonstrated that the resulting PDF uncertainties are competitive, though still larger, with those of NNPDF3.1. These considerations provide further weight for our default choice of the $H_{\mathrm{cut}}$ cut to the hadronic data.

#### The role of the $Q^2=2.7$ GeV$^2$ bin

We have stressed in Sect. 3 that, as opposed to NNPDF3.1, we include in the NNPDF3.1sx fits an additional low $Q^2$ bin of the inclusive HERA dataset, specifically the one with $Q^2=2.7$ GeV$^2$. This choice has the important advantage of extending the kinematic coverage of the fits from $x_{\mathrm{min}} \simeq 5 \times 10^{-5}$ down to $x_{\mathrm{min}} \simeq 3 \times 10^{-5}$. The main reason why this bin was excluded from previous NNPDF fits (as well as in most other global PDF fits) is its low value of $Q^2$, which lies at the boundary between perturbative and non-perturbative dynamics, and where fixed-order perturbation theory might not be fully appropriate. Here we show that this failure is not actually due to non-perturbative dynamics, but rather it represents a limitation of the fixed-order expansion in the small-*x* region enhanced by the larger value of $\alpha _s$. Indeed, we find that once NNLO fixed-order perturbation theory is supplemented by NLL*x* resummation, this bin can be described with similar quality as the rest of the HERA data.

**Fig. 19:**
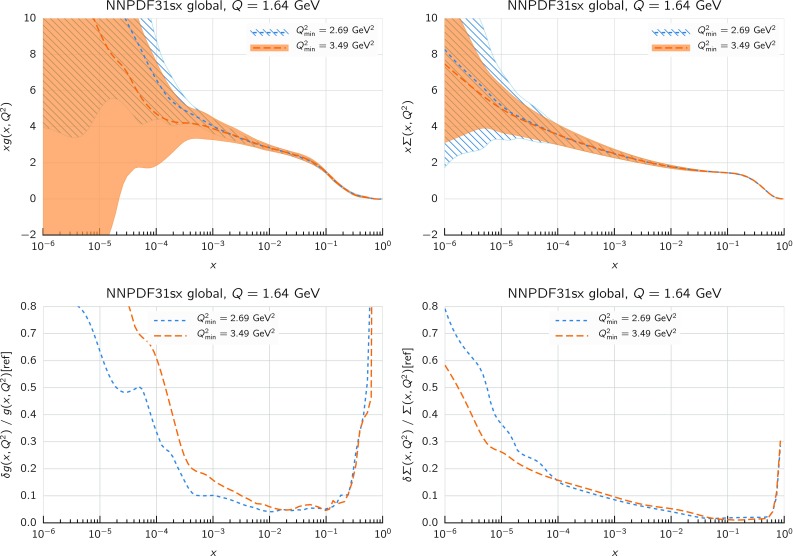
Comparison of the gluon (left) and quark singlet (right) at $Q_0=1.64$ GeV between the NNPDF3.1sx $\hbox {NNLO+NLL}x$ fits with the $Q_{\mathrm{min}}^2=2.69$ $\hbox {GeV}^2$ (baseline) and $Q_{\mathrm{min}}^2=3.49$ $\hbox {GeV}^2$ kinematic cuts (upper plots) together with the corresponding relative PDF uncertainties (lower plots)

To illustrate this point, we have computed the values of $\chi ^2/N_{\mathrm{dat}}$ for the $N_{\mathrm{dat}}=17$ data points that constitute the $Q^2=2.7$ GeV$^2$ bin of the inclusive HERA structure function dataset. We find that the values of $\chi ^2/N_{\mathrm{dat}}$ for this bin are 1.64 and 1.34 in the NNPDF3.1sx $\hbox {NLO+NLL}x$ and $\hbox {NNLO+NLL}x$ fits. These results can be compared with the corresponding values in the NLO and NNLO fits, which turn out to be 2.04 and 3.04, respectively. The trend is the same as that for the total NC HERA inclusive dataset (see Table 4), namely with the $\hbox {NNLO+NLL}x$ (NNLO) fit leading to the best (worst) overall description, and with the NLO and $\hbox {NLO+NLL}x$ values in between. Interestingly, we also see that for this specific fit $\hbox {NNLO+NLL}x$ theory leads to a rather better $\chi ^2$ than the $\hbox {NLO+NLL}x$ one, although the small number of data points prevents drawing any strong conclusion from this observation.

Once we have established that the fit quality to the $Q^2=2.7$ GeV$^2$ HERA bin is satisfactory when NLL*x* resummation is included, we can next turn to study the constraints that this bin has on the small-*x* PDFs. With this motivation, we have performed a global fit at $\hbox {NNLO+NLL}x$ with the same settings as the NNPDF3.1sx baseline but raising the low $Q^2$ kinematic cut from $Q_{\mathrm{min}}^2=2.69$ GeV$^2$ to $Q_{\mathrm{min}}^2=3.49$ GeV$^2$, as in NNPDF3.1, so that the HERA bin with $Q^2=2.7$ GeV$^2$ is excluded. In the latter case, the lowest HERA bin included is the one with $Q^2=3.5$ GeV$^2$.

The results are shown in Fig. 19, where the gluon and the quark singlet PDFs obtained in the NNPDF3.1sx $\hbox {NNLO+NLL}x$ fits with and without this additional bin are compared at the input parametrization scale of $Q_0=1.64$ GeV, together with their relative PDF uncertainties. We find that the inclusion of this extra $Q^2$ bin leads to a significant reduction of small-*x* uncertainty of the gluon in the region which is constrained by the data ($x\gtrsim 10^{-5}$), while the quark singlet is essentially unaffected. These results illustrate how the use of an improved theory, $\hbox {NNLO+NLL}x$ in this case, can lead indirectly to a decrease of the PDF uncertainties due to the possibility of including more data in the fits from a wider kinematic range.

## Small-*x* resummation and HERA structure functions

The results of the previous section provided two main pieces of information. First of all, the inclusion of small-*x* resummation improves the description of those datasets which represent the best probe of the small-*x* region, namely the inclusive and charm HERA structure functions. Second, the impact of resummation at the level of PDFs can be sizable. In this section, we focus on the HERA data in the small-*x* and small-$Q^2$ region, in order to further quantify the improvement in its description when fixed-order theory is supplemented by NLL*x* resummation.

We first compare the HERA structure functions at low *x* with fixed-order and resummed theoretical predictions, both for the inclusive and charm reduced cross-sections as well as for the longitudinal structure function $F_L$. In all cases, we highlight the improved description that is achieved once $\hbox {NNLO+NLL}x$ theory is used in all cases. To quantitatively investigate the evidence for the onset of small-*x* resummation in the HERA data, we introduce several estimators building upon the set of diagnostic tools first presented in Refs. [65, 66]. We finally study how removing HERA data at low-*x* and low-$Q^2$ affects global NNLO fits, and we discuss how the resulting PDFs are modified at medium- and large-*x*. This way, it is possible to assess whether the inclusion of data poorly described in a fixed-order analysis might be a source of bias for high-$Q^2$ phenomenology.

**Fig. 20:**
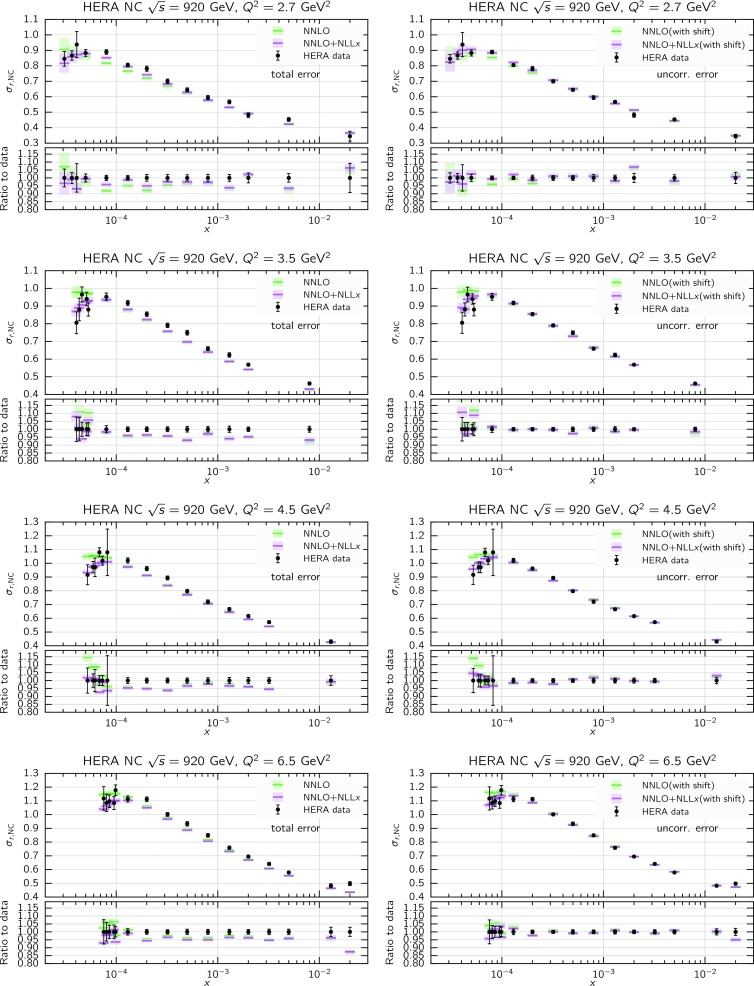
Comparison between the HERA NC reduced cross-section from the $\sqrt{s}=920$ GeV dataset and the results of the NNLO and $\hbox {NNLO+NLL}x$ fits with the corresponding PDF uncertainties. We show the results for the first four bins in $Q^2$ above the $Q^2_{\mathrm{min}}$ kinematic cut. For each bin we also show in the bottom panel the ratio of the theory predictions to the experimental data. The plots on the right show the theoretical prediction including the shifts as discussed in the text

### The HERA data in the small-*x* region

In order to investigate in greater detail how well resummed theory describes the low-$Q^2$ HERA cross-sections, we first perform a comparison of the theoretical predictions obtained using the results of the NNPDF3.1sx NNLO and $\hbox {NNLO+NLL}x$ global fits to the experimental data. To begin with, in Fig. 20 we show the neutral-current (NC) reduced cross-section, defined as

5.1 $$\begin{aligned} \sigma _{r,\mathrm{NC}}(x,Q^2,y)\equiv & {} {{\mathrm{d}^2\sigma _{\mathrm{NC}}}\over {\mathrm{d}x \mathrm{d}Q^2}}\cdot {{Q^4x}\over {2\pi \alpha Y_+}}\nonumber \\= & {} F_2(x,Q^2)-{{y^2}\over {Y_+}}F_L(x,Q^2), \end{aligned}$$

where $Y_+=1+(1-y)^2$ and $y={{Q^2}\over {sx}}$ is the inelasticity. This comparison is performed for the first four bins in $Q^2$ above our $Q_{\mathrm{min}}^2$ kinematic cut of the $\sqrt{s}=920$ GeV dataset, corresponding to $Q^2=2.7, 3.5, 4.5$ and 6.5 GeV$^2$ respectively. In the left plots, the uncertainty of the experimental data points is given by the sum in quadrature of the various sources of uncorrelated and correlated uncertainties, whereas the theoretical predictions include the associated PDF uncertainty. In the right plots, instead, only the uncorrelated uncertainties are shown in the data, and the correlations are taken into account via shifts which modify the theoretical prediction [187] and facilitate the graphical comparison. Note that these correlations are included in the $\chi ^2$ definition. However, unlike in a Hessian approach, in a Monte Carlo method one does not determine the best-fit systematic shifts. Rather, here we have computed them a posteriori, under the assumption that the uncertainties are gaussian, which is not necessarily true in a Monte Carlo fit. Therefore, this comparison must be interpreted with care.

From this comparison, we see that for $x\gtrsim 5\times 10^{-4}$ the results of the NNLO and $\hbox {NNLO+NLL}x$ fits are essentially identical; in both cases, the theoretical predictions undershoot the data. The trend changes for values of *x* smaller than $5\times 10^{-4}$, where the NNLO and the $\hbox {NNLO+NLL}x$ predictions start to differ. Around this value, we observe that the reduced cross-section exhibits a slope change too: the data stop rising and, after a turnover, the reduced cross-section starts decreasing. As a result, the NNLO prediction starts to overshoot the data, whereas the $\hbox {NNLO+NLL}x$ prediction is in reasonable agreement with the data for $x \lesssim 10^{-4}$. It is worth observing that the differences between the NNLO and $\hbox {NNLO+NLL}x$ predictions are relatively small and concern only a limited number of points. By looking at the bottom panels in Fig. 20, where we show the ratio to the experimental data, we see that the two predictions differ by at most $10\%$ and only for the smallest values of *x*. Yet the combined HERA dataset is so precise that the improvement in the description provided by small-*x* resummation is clearly visible at the $\chi ^2$ level, as was shown in Table 4, and will be discussed further below in Sect. 5.2.

The improved description of the inclusive reduced cross-section data at small *x* can be in part traced back to the role of the longitudinal structure function $F_L(x,Q)$. As reviewed in Sect. 2, $F_L$ is particularly sensitive to the effects of small-*x* resummation, and in particular to deviations from the DGLAP framework. The reason is that it vanishes at the Born level, and therefore it receives gluon-initiated contributions already at its first non-trivial order. As shown in Fig. 5, the differences between the NNLO and $\hbox {NNLO+NLL}x$ can be as large as $\sim 30\%$ at the lowest *x* and $Q^2$ bins for which there are data available. As a consequence, at small *x* and small $Q^2$ the contribution of $F_L$ to $\sigma _{r,\mathrm NC}$ can be significant, see Eq. (5.1), thus partly explaining the differences between the NNLO and $\hbox {NNLO+NLL}x$ predictions observed in Fig. 20. Therefore, it is useful to compare the predictions also for the longitudinal structure function $F_L$ in the NNLO and $\hbox {NNLO+NLL}x$ fits.

**Fig. 21:**
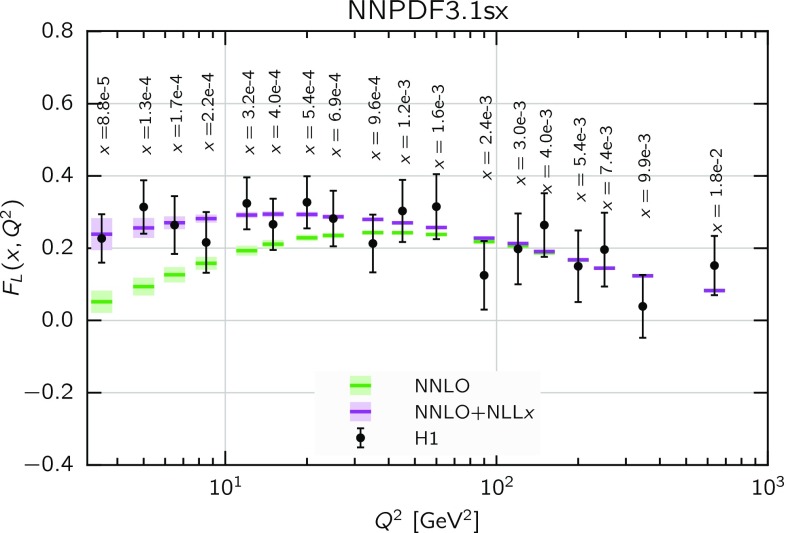
The longitudinal structure function $F_L(x,Q^2)$ as a function of $Q^2$ for different *x* bins for the most recent H1 measurement [188], comparing the results of the NNLO and $\hbox {NNLO+NLL}x$ fits

In Fig. 21 we compare the latest measurements of $F_L$ from the H1 collaboration [188]^5^ with the predictions from the NNPDF3.1sx NNLO and $\hbox {NNLO+NLL}x$ fits. Note that our fits already include the constraints from $F_L$, not directly but rather via its contribution to the NC reduced cross-section, Eq. (5.1). In this comparison, the experimental uncertainties have been added in quadrature, and each value of $Q^2$ corresponds to a different *x* bin as indicated in the plot. The NNPDF3.1sx results are shown down to the smallest scale for which one can reliably compute a prediction,^6^ which is set by the initial parametrization scale $Q_0^2=2.69$ GeV$^2$.

We see that for $Q^2 \lesssim 100$ GeV$^2$ there are significant differences between the $\hbox {NNLO+NLL}x$ and the NNLO predictions, which can be traced back to a combination of the corresponding differences for the input small-*x* gluon and those in the splitting and coefficient functions (see Fig. 5). The $\hbox {NNLO+NLL}x$ result is larger than the NNLO result by a significant amount: at $Q^2 \simeq 10$ GeV$^2$, the resummed calculation is more than a factor 2 larger than the NNLO result. Moreover, while at NNLO $F_L$ starts becoming negative at small *x* and $Q^2$ (below the scale where the positivity constraints are imposed in the NNPDF fits) the $\hbox {NNLO+NLL}x$ result instead exhibits a flat behavior even for the smallest values of $Q^2$. The larger value of $F_L$ with the $\hbox {NNLO+NLL}x$ theory leads to a lower reduced cross-section at high *y*, with a more pronounced turnover, thus giving a better description of $\sigma _{r,\mathrm NC}$ at small *x*, as shown in Fig. 20.

**Fig. 22:**
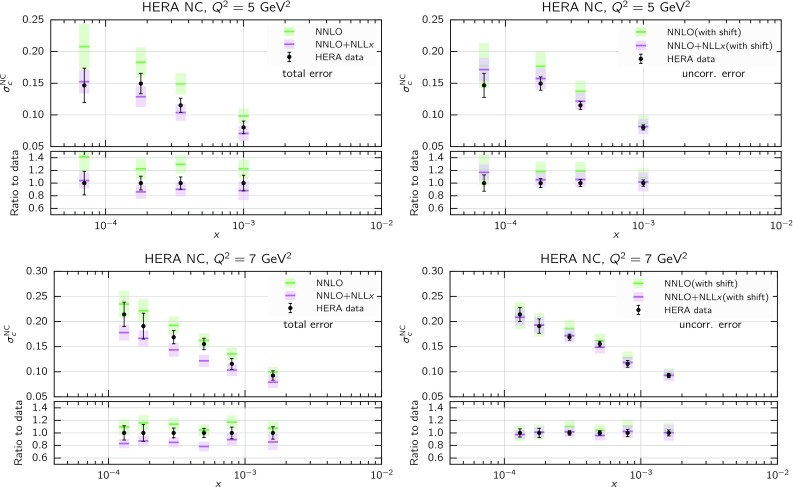
Same as Fig. 20 for the HERA charm-production cross-sections

Finally, in Fig. 22 we show a similar comparison to that of Fig. 20, this time for the HERA charm-production reduced cross-sections. Here we also show the two $Q^2$ bins about the lower $Q^2_{\mathrm{min}}$ cut, which in this case correspond to the $Q^2=5$ and 7 GeV$^2$ bins. We find that especially for the bin with $Q^2=5$ GeV$^2$, the NNLO+NNL*x* prediction agrees well with the HERA data while the NNLO one overshoots it. We remind the reader again that these graphical comparisons do not take into account the correlations between systematic uncertainties. The large difference between the $\chi ^2$ at NNLO and at $\hbox {NNLO+NLL}x$ is therefore only partially captured by Fig.  22. As we shall see in greater detail in Sect. 5.2, also in this case the deterioration of the NNLO $\chi ^2$ with respect to the NNLO+NNL*x* result shown in Table 4 stems mostly from the low-$Q^2$, low-*x* bins.

Note that the HERA charm cross-sections are extracted from the experimentally measured fiducial cross-section [133] by extrapolation to the full phase space using the fixed-order $\mathcal {O}( \alpha _s^2)$ calculation of the HVQDIS program [190], based on the fixed-flavor number scheme. This should be contrasted with the inclusive neutral-current structure function measurements, which are determined from the outgoing lepton kinematics and therefore do not assume any theory input. Given that we have shown that fixed-order and resummed predictions for $F_2^c$ can exhibit important differences at small *x*, such theory-based extrapolation based on the $\mathcal {O}(\alpha _s^2)$ fixed-order calculation might introduce a bias whose size is difficult to quantify. It is quite possible that a more consistent analysis of the raw data based instead on an extrapolation using resummed theoretical predictions might further improve the already good agreement of the extracted charm cross-section with the $\hbox {NNLO+NLL}x$ fit.

**Fig. 23:**
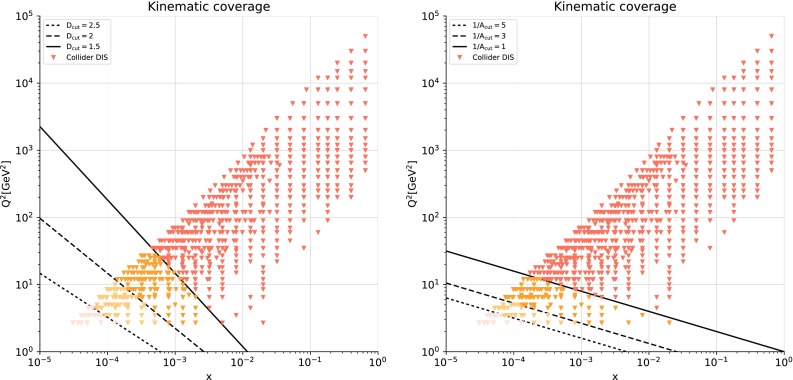
The kinematic coverage of the HERA inclusive structure function data that enters the NNPDF3.1sx fits. The tilted lines represent representative values of the cut to DIS data applied after the fit to study evidence for BFKL effects at small *x* and small $Q^2$. Left plot: perturbative-inspired cut Eq. (5.2); right plot: saturation-inspired cut Eq. (5.4). Note that the data points affected by the various cuts are plotted with different shades

### Quantifying the onset of small-*x* resummation in the HERA data

In this section we resort to a number of statistical estimators to identify more precisely the onset of small-*x* resummation in the inclusive and charm HERA measurements. First, we perform a detailed $\chi ^2$ analysis, which we then complement by a study of the pulls between theory and HERA data.

#### $\chi ^2$ analysis

The $\chi ^2/N_{\mathrm{dat}}$ values summarized in Table 4 indicate that the fit quality of the inclusive HERA structure functions improves when resummation effects are included: this is particularly true at NNLO, where the total $\chi ^2$ drops by $\Delta \chi ^2=-121$ units in the $\hbox {NNLO+NLL}x$ fit. We now want to identify the origin of this improvement, and investigate to what extent it arises from a better description of the data in the small-*x* and small-$Q^2$ region where the effects of small-*x* resummation are expected to be most important.

To achieve this goal, we have recomputed the $\chi ^2/N_{\mathrm{dat}}$ values of the HERA inclusive and charm cross-sections using the NNPDF3.1sx NLO, NNLO, $\hbox {NLO+NLL}x$, and $\hbox {NNLO+NLL}x$ global fits with the default choice $H_{\mathrm{cut}}=0.6$, excluding those data points for which

5.2 $$\begin{aligned} \alpha _s(Q^2) \ln {{1}\over {x}} \ge D_{\mathrm{cut}}. \end{aligned}$$

The condition Eq. (5.2) is designed to exclude data for which the small-*x* logarithmic terms are expected to be of the same size at all orders in the coupling $\alpha _s$, thus potentially spoiling the perturbative behavior of the theoretical predictions at fixed order.

From basic considerations (see also Sect. 3.2), one would expect fixed-order perturbation theory to break down for $\alpha _s(Q^2) \ln {{1}\over {x}}$ of order 1. The parameter $D_{\mathrm{cut}}$ should thus be of order 1 as well. By varying the value of $D_{\mathrm{cut}}$, we can vary the number of data points excluded from the computation of the $\chi ^2/N_{\mathrm{dat}}$. For sufficiently small values of $D_{\mathrm{cut}}$, all contributions which potentially spoil perturbation theory should be cut away, and we should thus find that small-*x* resummation does not improve the quality of the fit. Then as we increase $D_{\mathrm{cut}}$, more data points at small *x* and $Q^2$ will be included, and the effects of the resummation should become apparent. A kinematic plot showing the HERA structure function data which are cut for various values of $D_{\mathrm{cut}}$ is shown in the left panel of Fig. 23. We emphasize that this cut should not be confused with the $H_{\mathrm{cut}}$ cut defined in Eq. (3.1), which was used to determine which hadronic data enter in the fit; here the parameter $D_{\mathrm{cut}}$ applies only to DIS structure functions and is used as an a posteriori diagnosis tool after the fit has been performed.

**Fig. 24:**
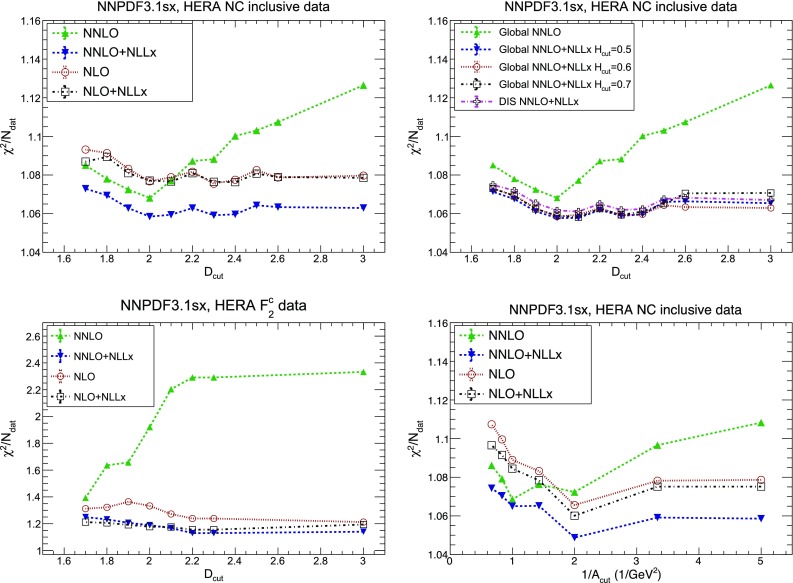
Upper left: the values of $\chi ^2/N_{\mathrm{dat}}$ in the NNPDF3.1sx global fits for the HERA NC inclusive structure function data for different values of the cut $D_{\mathrm{cut}}$ Eq. (5.2), comparing the results of the NLO, $\hbox {NLO+NLL}x$, NNLO, and $\hbox {NNLO+NLL}x$ fits. Upper right: same comparison, now between the global NNLO and $\hbox {NNLO+NLL}x$ baseline fits with the $\hbox {NNLO+NLL}x$ global fits with $H_{\mathrm{cut}}=0.5$ and 0.7 and with the DIS-only fit. Bottom left: same as above for the HERA charm-production data. Bottom right: same as upper left, now with the saturation-inspired cut Eq. (5.4)

In Fig. 24 we display the values of $\chi ^2/N_{\mathrm{dat}}$ for the HERA neutral-current inclusive (top left) and charm (bottom left) reduced cross-sections as a function of $D_{\mathrm{cut}}$. First of all, we observe that at NNLO the $\chi ^2/N_{\mathrm{dat}}$ increases sharply for $D_{\mathrm{cut}}\gtrsim 2$, or, equivalently, as more data from the small-*x* and small-$Q^2$ region are included, both for the inclusive and the charm data. On the other hand, this trend disappears for the $\hbox {NNLO+NLL}x$ fits: in this case the value of $\chi ^2/N_{\mathrm{dat}}$ is flat for all $D_{\mathrm{cut}}$ values in the studied range.

Another interesting feature of these plots is that the stability with respect to the value of $D_{\mathrm{cut}}$ is also present for the NLO and $\hbox {NLO+NLL}x$ fits. Indeed, the $\chi ^2/N_{\mathrm{dat}}$ values for the NLO, $\hbox {NLO+NLL}x$, and $\hbox {NNLO+NLL}x$ fits all exhibit a rather similar shape. This is of course a consequence of the fact that, as shown in Sect. 4, the PDFs obtained from the fits using these three theories are rather close to each other, whereas the NNLO PDFs are very different at small *x*. Remarkably, for the inclusive data especially the $\hbox {NNLO+NLL}x$ fits lead to a better $\chi ^2/N_{\mathrm{dat}}$ than the NLO and $\hbox {NLO+NLL}x$ ones, presumably due to the additional NNLO corrections included in the $\hbox {NNLO+NLL}x$ matched calculations. This result highlights the importance of the NNLO corrections for the optimal description of the medium and large-*x* HERA data.

The results of Fig. 24 demonstrate that fixed-order NNLO theory does not provide a satisfactory description of either the inclusive or charm DIS data at small *x* and small $Q^2$. The better description is instead achieved by including NLL*x* effects, providing direct evidence of the need for small-*x* resummation at small *x*. Moreover, we observe that the rise in the $\chi ^2/N_{\mathrm{dat}}$ values of the NNLO fits becomes very significant for $D_{\mathrm{cut}}\gtrsim 2$. This means that BFKL effects at NNLO approximately start to become important when

5.3 $$\begin{aligned} \ln {{1}\over {x}} \gtrsim 1.2 \ln {{Q^2}\over {\Lambda ^2}}, \end{aligned}$$

see Eq. (3.2), which implies, for instance, that the effects of small-*x* resummation become phenomenologically relevant around $x\simeq 8\times 10^{-4}$ ($2.7\times 10^{-4}$) for $Q^2=2.7$ GeV$^2$ (6.5 GeV$^2$). This estimate is consistent with the results presented in Sects. 2 and 4.

**Fig. 25:**
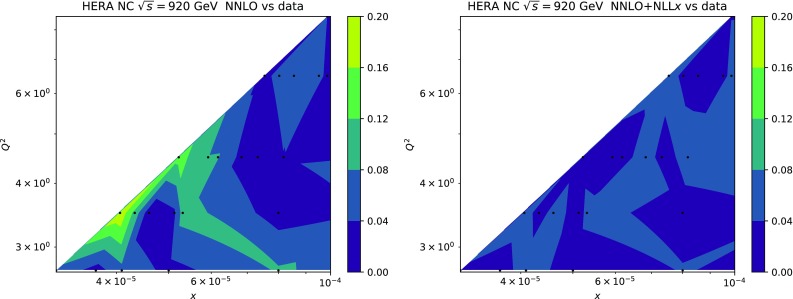
Left panel: interpolated representation of the relative pull Eq. (5.5) between the HERA NC reduced cross-section data at $\sqrt{s}=920$ GeV and the NNLO fit, in the small-*x* and small-$Q^2$ region. Right panel: same as the left panel now for the $\hbox {NNLO+NLL}x$ fit

To study whether the treatment of the hadronic data in the PDF fits can modify this conclusion, in the upper right panel of Fig. 24 we also compare the $\chi ^2/N_{\mathrm{dat}}$ values as a function of $D_{\mathrm{cut}}$ for the NNPDF3.1sx $\hbox {NNLO+NLL}x$ global fits with the three $H_{\mathrm{cut}}$ values discussed in Sect. 4.2.3, namely $H_{\mathrm{cut}}=0.5$, 0.6 and 0.7, as well as with the global $H_{\mathrm{cut}}=0.6$ NNLO fit and the $\hbox {NNLO+NLL}x$ DIS-only fit. These comparison illustrate that our quantitative conclusions are to a very good approximation independent of the specific cut applied to the hadronic data: very similar $\hbox {NNLO+NLL}x$ results are found in the global fit irrespective of the value of $H_{\mathrm{cut}}$, as well as for the corresponding DIS-only fit. We have also verified that the same conclusion holds for the NLO and $\hbox {NLO+NLL}x$ fits.

In Refs. [65, 66], a similar cutting exercise was performed, but in that case the specific form of the cut to the small-*x* and small-$Q^2$ data was inspired by saturation arguments. Specifically, the condition used to exclude data points was

5.4 $$\begin{aligned} Q^2 x^{\lambda } \ge A_{\mathrm{cut}} , \end{aligned}$$

with $\lambda =0.3$. The value of $A_{\mathrm{cut}}$ determines how stringent is the cut: the larger its value, the more data points excluded (so $1/A_{\mathrm{cut}}$ behaves qualitatively in the same way as $D_{\mathrm{cut}}$). While the inspiration for the cut Eq. (5.4) is different from that of Eq. (5.2) (which is based instead on perturbative considerations), the practical result is the same, with only some differences on the exact shape of the cut in the $(x,Q^2)$ plane (see the right panel of Fig. 23). The results for the $\chi ^2/N_{\mathrm{dat}}$ as a function of $1/A_{\mathrm{cut}}$ are shown in the bottom right panel of Fig. 24, and indeed confirm that the trend is essentially the same, irrespectively of the specific details of how the small-*x* and $Q^2$ data are cut.

In summary, the results collected in Fig. 24 clearly demonstrate the onset of BFKL dynamics in the small-*x* and $Q^2$ region for both the inclusive and charm HERA data. Specifically, we find that the use of $\hbox {NNLO+NLL}x$ theory gives the best description of the HERA data in the small-*x* region, while NNLO theory gives a significantly worse description. Moreover, our results also allow us to determine the kinematic region where small-*x* resummation effects start to become phenomenologically relevant, thus providing useful guidance to estimate their reach at the LHC as well as for future colliders.

#### Pull analysis

A complementary approach to further investigating the onset of BFKL dynamics in the low-*x* region, and to make connection with the analysis of Refs. [65, 66], is provided by the calculation of the relative pull between experimental data and theory. This relative pull is defined as

5.5 $$\begin{aligned} P_i^{\mathrm{rel}}(x,Q^2)\equiv {{\big |\sigma _{\mathrm{data},i}-\sigma _{\mathrm{th},i}\big |}\over { \left( \sigma _{\mathrm{data},i}+\sigma _{\mathrm{th},i}\right) /2 }}, \end{aligned}$$

where the normalization is given by the average of central values.

This estimator allows us to quantify the absolute size of the differences between data and theory in units of the cross-section itself. Here we focus on the results computed with NNLO and $\hbox {NNLO+NLL}x$ theory, using the NNPDF3.1sx sets obtained in the respective global fits with the default cut $H_{\mathrm{cut}}=0.6$.

To visualize the differences between data and theory in the small-*x* and small-$Q^2$ region, we can represent the relative pull $P_i^{\mathrm{rel}}(x,Q^2)$, Eq. (5.5), as a function of (*x*, $Q^2$) in the relevant region of the kinematic plane. In Fig. 25 we show an interpolated representation of $P^{\mathrm{rel}}(x,Q^2)$ for the HERA neutral-current dataset at $\sqrt{s}=920$ GeV and the NNLO and $\hbox {NNLO+NLL}x$ fits. In the case of the NNLO fit, the relative differences between theory and data can be up to $\sim 20\%$ at small-*x* and $Q^2$, and reduce to less than a few percent at larger *x* or $Q^2$. On the other hand, the agreement between data and theory is markedly improved in the case of the $\hbox {NNLO+NLL}x$ fit: the quality of the data description is essentially the same everywhere in the region considered, and the relative differences between data and theory are everywhere below the $8\%$ level. These plots show that by using $\hbox {NNLO+NLL}x$ theory, one can achieve a satisfactory description of the inclusive HERA measurements in the entire region spanned by the available data.

**Fig. 26:**
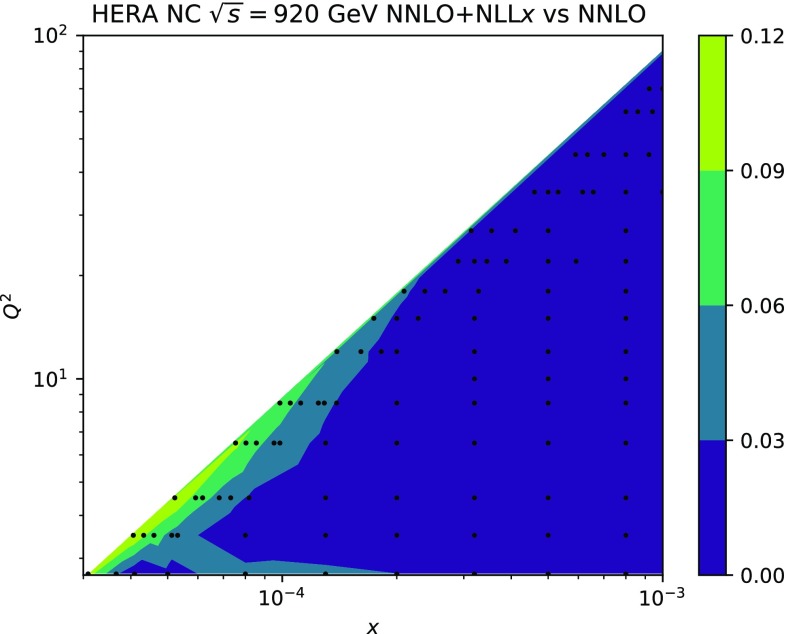
Same as Fig. 25, now for the relative difference in the theoretical predictions of the HERA reduced cross-sections between the NNLO and $\hbox {NNLO+NLL}x$ fits, Eq. (5.6). Note the different color code and *x* and $Q^2$ ranges with respect to Fig. 25

**Fig. 27:**
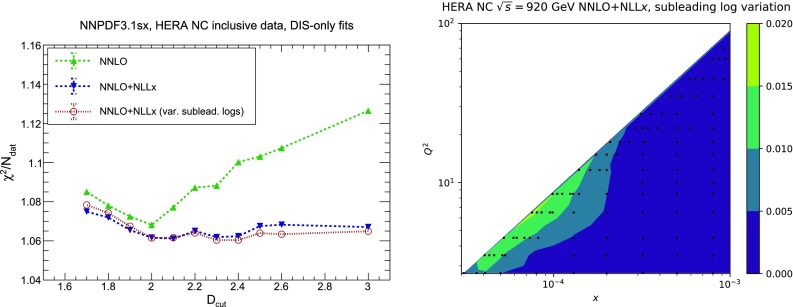
Left panel: the values of $\chi ^2/N_{\mathrm{dat}}$ in the NNPDF3.1sx DIS-only fits for the HERA NC inclusive structure function data for different values of the cut $D_{\mathrm{cut}}$ Eq. (5.2), compared to the results of a fit where the subleading logarithms are varied. Right panel: same as Fig. 26, now for the relative difference between the $\hbox {NNLO+NLL}x$ DIS-only fit and the $\hbox {NNLO+NLL}x$ DIS-only fit performed with a variation of subleading logarithms. Note the different color code with respect to Fig. 26

In order to further quantify differences and similarities between the NNLO and $\hbox {NNLO+NLL}x$ theoretical predictions, in Fig. 26 we show a similar relative pull as in Eq. (5.5), now between the theoretical predictions for the HERA reduced cross-sections obtained with the NNLO and the $\hbox {NNLO+NLL}x$ theory and fits, namely

5.6 $$\begin{aligned} \widetilde{P}_i^{\mathrm{rel}}(x,Q^2)\equiv {{ \big |\sigma ^{{\mathrm{NNLO+NLL}}x}_{\mathrm{th},i} - \sigma ^{\mathrm{NNLO}}_{\mathrm{th},i} \big | }\over { \left( \sigma ^{{\mathrm{NNLO+NLL}}x}_{\mathrm{th},i} + \sigma ^{\mathrm{NNLO}}_{\mathrm{th},i}\right) /2 }}. \end{aligned}$$

Note that in this comparison both the color code and the $(x,Q^2)$ ranges are different from those of Fig. 25. From the results of Fig. 26 we see that the differences between the cross-sections computed with NNLO and $\hbox {NNLO+NLL}x$ theory are between 5% and 10% for $Q^2 \lesssim 10$ GeV$^2$ and $x\lesssim 2\times 10^{-4}$. Once we move away from this region, differences become smaller. For $x \gtrsim 2 \times 10^{-4}$, we find that the differences are always smaller than $\sim 3\%$, for any value of $Q^2$. This comparison provides a detailed snapshot of the region in the $(x,Q^2)$ plane where the impact of resummation is phenomenologically more relevant, and is consistent with the results shown in Fig. 20 and the conclusions of the $\chi ^2$ profile analysis of Sect. 5.2.1.

#### Sensitivity to subleading logarithms

Finally, we can study if our conclusions are stable with respect to variations of unknown subleading logarithms. To this end, in the left panel of Fig. 27 we show the values of $\chi ^2/N_{\mathrm{dat}}$ for the HERA NC inclusive reduced cross-section as a function of $D_{\mathrm{cut}}$, now comparing the DIS-only fit at NNLO and $\hbox {NNLO+NLL}x$ to the $\hbox {NNLO+NLL}x$ fit where subleading logarithms are introduced as described in Sect. 4.1. We observe that the two $\hbox {NNLO+NLL}x$ profiles are very similar, with the $\chi ^2/N_{\mathrm{dat}}$ of the alternative fit being slightly lower at larger $D_{\mathrm{cut}}$. To better quantify the differences between the two variants, in the right panel of Fig. 27 we also show the relative pull Eq. (5.6) between the theoretical predictions for the two $\hbox {NNLO+NLL}x$ fits for the HERA neutral current $\sqrt{s}=920$ GeV dataset. We observe that the relative difference is at most $2\%$ for the smallest values of *x* and $Q^2$ probed by the dataset, and is below $0.5\%$ for all $x \gtrsim 3\times 10^{-4}$, independently of the value of $Q^2$. This analysis shows that our results are stable with respect to variations of subleading logarithms.

**Fig. 28:**
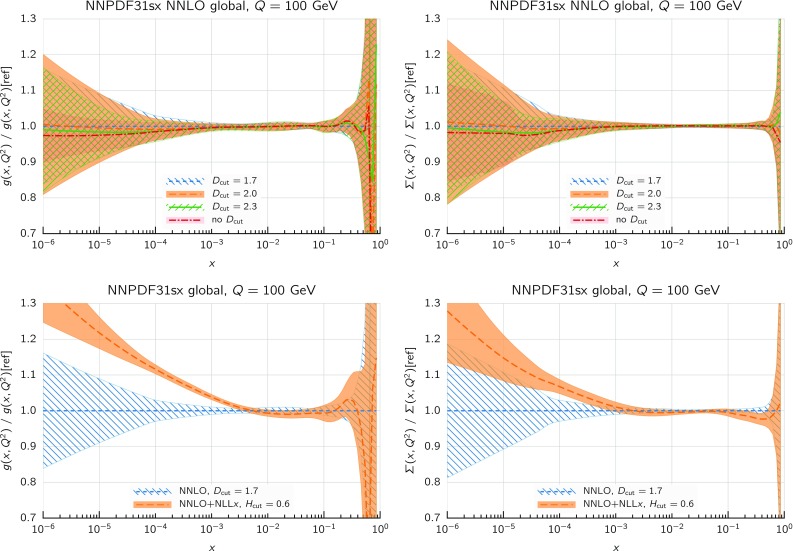
Upper plots: comparison between the gluon and quark singlet at $Q=100$ GeV from the NNPDF3.1sx NNLO fits with various values of $D_{\mathrm{cut}}$ with the corresponding fit without that kinematic cut. Bottom plots: comparison between the NNLO fit with $D_{\mathrm{cut}}=1.7$ and the baseline $\hbox {NNLO+NLL}x$ fit (with $H_{\mathrm{cut}}=0.6$). Both comparisons are shown normalized to the central value of the $D_{\mathrm{cut}}=1.7$ fit

### Impact of the small-*x* HERA data on PDFs at medium and large-*x*

In the last part of this section, we present results of additional NNPDF3.1sx NNLO fits where we have *removed* a number of HERA structure function data points in the small-*x* and $Q^2$ region, in order to study how the resulting PDFs are affected by the use of such reduced dataset. This exercise allows us to understand to what extent existing NNLO global PDF fits might be biased by fitting low-*x* data while neglecting the effects of small-*x* resummation. Since we have just demonstrated that at small *x* the HERA structure functions prefer $\hbox {NNLO+NLL}x$ theory to the NNLO one, it may be advisable to apply dedicated kinematic cuts in the small-*x* and $Q^2$ region in standard NNLO analyses. This would ensure on one hand that the fitting dataset corresponds to a region where a fixed-order perturbative expansion is reliable, and on the other hand that the estimate of the uncertainties at small *x* is more reliable.

For this purpose, we have performed variants of the NNPDF3.1sx NNLO global fit without any cut on the hadronic data (that is, $H_{\mathrm{cut}}=\infty$) but where instead we impose the cut Eq. (5.2) to the DIS structure function data *before* fitting, thus reducing the number of data points in the small-*x* and small-$Q^2$ region. Specifically, we have performed NNLO fits with $D_{\mathrm{cut}}=1.7,\, 2.0$ and 2.3, as a well as a fit without cutting any data ($D_{\mathrm{cut}}=\infty$) as a reference. The motivation for this range of $D_{\mathrm{cut}}$ values is the observation (see Fig. 24) that $D_{\mathrm{cut}}\simeq 2$ indicates the region where the effects of small-*x* resummation start to become significant.

The comparison between the NNPDF3.1sx NNLO fits with different cuts to the DIS data is shown in the upper plots of Fig. 28. Specifically, we show the comparison between the gluon and quark singlet from the fits with various values of $D_{\mathrm{cut}}$ with the corresponding fit without that cut ($D_{\mathrm{cut}}=\infty$) at $Q=100$ GeV. From this comparison we can see that – as expected – the higher the value of $D_{\mathrm{cut}}$, the smaller are the PDF uncertainties at small *x* due the increase in kinematic coverage of the fitted HERA data. However, the central values remain very stable, even at the lowest values of *x*. Additionally, we also see that for $x\gtrsim 5\times 10^{-4}$ the gluon and quark singlet are extremely stable with respect to the $D_{\mathrm{cut}}$ variations, both in terms of central value and of PDF uncertainties. Therefore, we conclude that current NNLO fits are not biased in the region relevant for precision LHC phenomenology, even if the fits include points at small *x*, while neglecting resummation effects.

It is also interesting to compare the NNPDF3.1sx NNLO fit with $D_{\mathrm{cut}}=1.7$ with our default $\hbox {NNLO+NLL}x$ global fit, namely the one with the cut in hadronic data corresponding to $H_{\mathrm{cut}}=0.6$, but $D_{\mathrm{cut}}=\infty$. This comparison allows us to understand if the PDF uncertainties of the conservative NNLO fits, where data points at small *x* and small $Q^2$ have been removed, account for the PDF shift induced by using the more accurate $\hbox {NNLO+NLL}x$ theory. We show this comparison in the bottom plots of Fig. 28 at the scale $Q=100$ GeV. Whereas for the quark singlet the shift between the $\hbox {NNLO+NLL}x$ and NNLO fits is mostly covered by the corresponding PDF uncertainties, for the gluon the shift in central values is bigger and is not covered by the PDF uncertainties, despite the larger PDF errors of the fit with the conservative dataset.

The results of Fig. 28 suggest that at small *x* the theoretical uncertainties associated with the NNLO gluon are comparable to or larger than the PDF uncertainties. Moreover, we observe that the shift induced by $\hbox {NNLO+NLL}x$ theory is covered by the PDF uncertainties only for values of *x* larger than $x\simeq 3\times 10^{-3}$. Therefore, for processes sensitive to the small-*x* region, including a number of LHC cross-sections, current NNLO PDF uncertainties do not fully account for the total theoretical uncertainty, suggesting that the use of $\hbox {NNLO+NLL}x$ theory would lead to more reliable theoretical predictions.

## Small-*x* phenomenology at the LHC and beyond

In this section we explore some of the phenomenological implications of the NNPDF3.1sx fits. First of all, we present a first assessment of the possible impact of NLL*x* resummation at the LHC. We then move to DIS-like processes, for which we can produce fully consistent $\hbox {NNLO+NLL}x$ predictions. In this context, we consider the implications of the NNPDF3.1sx fits for the ultra-high-energy (UHE) neutrino–nucleus cross-sections as well as for future high-energy lepton–proton colliders such as the LHeC [191] and the FCC-eh [192, 193], illustrating the key role that small *x* resummation could play in shaping their physics program.

### Small-*x* resummation at the LHC

In this section we perform a first exploration of the potential effects of small-*x* resummation on precision LHC phenomenology. We start by considering parton luminosities and then we estimate the effects of small-*x* resummation for electroweak gauge boson production at the LHC. The latter study will, however, be necessarily only qualitative, since as explained in Sect. 2 the relevant small-*x* resummed partonic cross-sections are not yet implemented in a format amenable for phenomenological applications. The studies of this subsection will thus be complementary to previous estimates of the effects of small-*x* resummation on inclusive LHC processes in which both evolution and cross-section were resummed, but the PDFs used were fixed rather than refitted [44, 185].

#### Parton luminosities

In order to provide a first insight on the possible impact of NLL*x* resummation effects on hadronic cross-sections, it is useful to consider its effects on the parton luminosities. We consider both the integrated parton luminosity Eq. (2.3) and also luminosities differential in rapidity (see e.g. [194])

6.1 $$\begin{aligned} {{\mathrm{d} \mathcal L_{ij}}\over {\mathrm{d}y}} \left( x,\mu ^2, y \right) = f_i \left( \sqrt{x} e^y,\mu ^2\right) f_j \left( \sqrt{x}e^{-y},\mu ^2 \right) . \end{aligned}$$

We assume the production of a hypothetical final state with invariant mass $$M_X$$, so that $$x=M_X^2/s$$ with $$\sqrt{s}$$ being the LHC center-of-mass energy, and we take the factorization scale to be $$\mu ^2=M_X^2$$. Despite offering only a qualitative estimate of the effects of small-*x* resummation, parton luminosities contain the bulk of the information as regards the partonic contributions to a given process. In particular, rapidity-dependent PDF luminosities provide a direct mapping between regions in the $$(x,Q^2) $$ plane (PDF sensitivity) and those in the $$\left( M_X,y\right) $$ plane (kinematic coverage for collider production), assuming leading-order production kinematics.

**Fig. 29:**
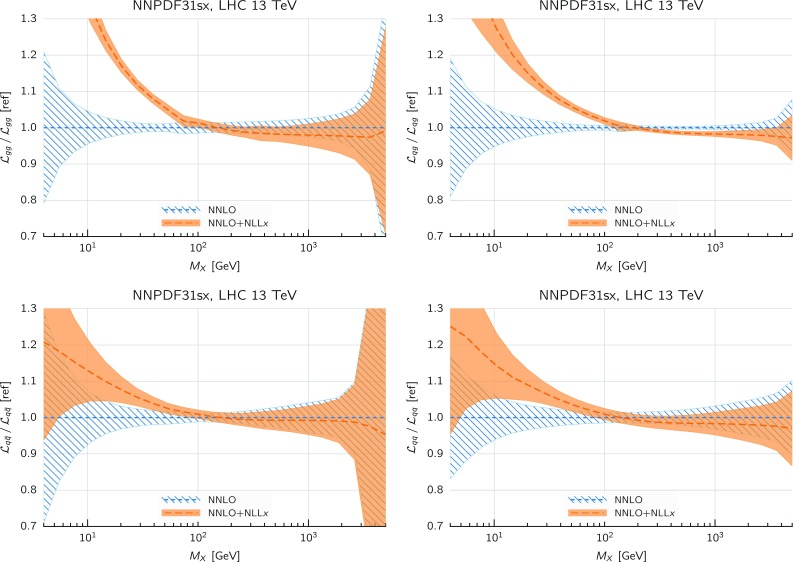
The gluon–gluon, quark–gluon, quark–antiquark and quark–quark PDF luminosities, Eq. (2.3), at $\sqrt{s}=13$ TeV as a function of the final-state invariant mass $M_X$, comparing the NNPDF3.1sx NNLO and $\hbox {NNLO+NLL}x$ global fits

Let us start with the integrated parton luminosities, Eq. (2.3). In Fig. 29 we show the gluon–gluon, quark–gluon, quark–antiquark and quark–quark PDF luminosities at $\sqrt{s}=13$ TeV as a function of the invariant mass $M_X$, comparing the NNPDF3.1sx global fits based on NNLO and $\hbox {NNLO+NLL}x$ theory respectively.

For the two gluon-initiated luminosities, the effects are very large for $M_X \lesssim 100$ GeV, and smaller above that value.

For the *gg* luminosity, for instance, the ratio between the NNLO and $\hbox {NNLO+NLL}x$ results can be more than $\sim 30\%$ for $M_X \simeq 10$ GeV, a region relevant for instance for open *B*-meson production.

Even larger effects can be expected for processes at smaller invariant masses, such as *D*-meson or $J/\Psi$ production.

At $M_X \gtrsim 100$ GeV, a region relevant for e.g. top-quark pair production, the gluon-induced luminosities are instead reduced, albeit by only a few per cent.

In the case of the quark–antiquark and quark–quark luminosities, the differences due to resummation are less significant, with the NNLO and $\hbox {NNLO+NLL}x$ luminosities in agreement within PDF uncertainties for the entire $M_X$ range.

However, the effects of small-*x* resummation are not negligible; for instance, they could still be as large as 10% at $M_X=10$ GeV, a region probed by the LHC in processes such as low-mass Drell–Yan production.

At larger invariant masses the differences between NNLO and $\hbox {NNLO+NLL}x$ are again down to 1–2%.

Thus, in this region the effects are small, but nevertheless of the same order as the experimental uncertainties of recent high-precision LHC measurements, such as for instance the ATLAS 2011 *W*, *Z* rapidity distributions [152] or the CMS *Z* $p_T$ distributions [169].

It is important to emphasize here that the luminosity comparison in Fig. 29 provides only a rough estimate of the actual differences between the NNLO and the fully resummed $\hbox {NNLO+NLL}x$ cross-sections, since a quantitative assessment requires the resummation of the partonic cross-sections for the relevant processes, and this can be as large as the difference in the luminosities [44, 185].

This said, the results of Fig. 29 show that the effects of small-*x* resummation are potentially significant for LHC cross-sections, in particular for those with large gluon-initiated contributions.

**Fig. 30:**
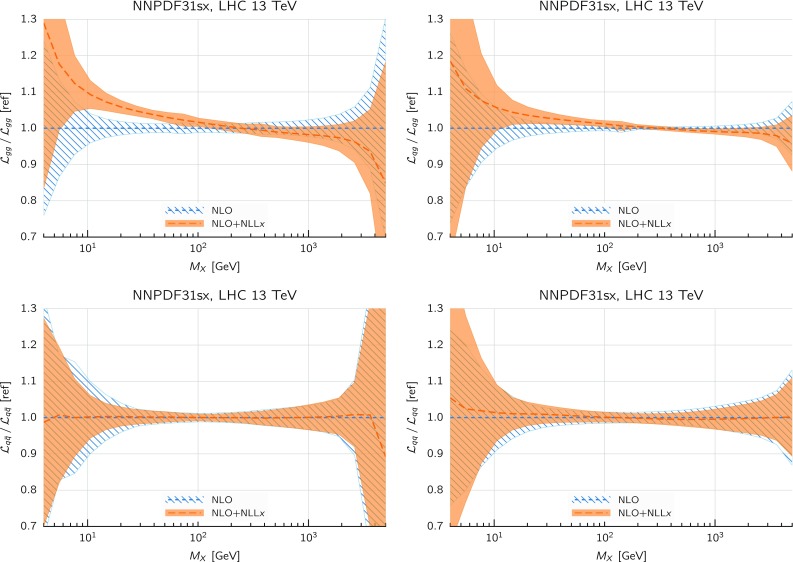
Same as Fig. 29, now comparing the results of the $\hbox {NLO+NLL}x$ and NLO fits

Next, in Fig. 30 we show same comparison but this time between the NLO and $\hbox {NLO+NLL}x$ fits. As discussed in Sect. 4, we expect the differences to be more moderate compared to the NNLO fits case. Indeed, the differences are now much smaller, both for the gluon-initiated and for the quark-initiated luminosities. The most significant effect of resummation can again be seen in the *gg* luminosity, but now only at the $10\%$ level at $M_X\lesssim 10$ GeV. The other luminosities all agree within uncertainties. Henceforth, we will focus on the comparison between the $\hbox {NNLO+NLL}x$ and the NNLO fits, as in all cases the corresponding differences between $\hbox {NLO+NLL}x$ and NLO would always be much smaller.

**Fig. 31:**
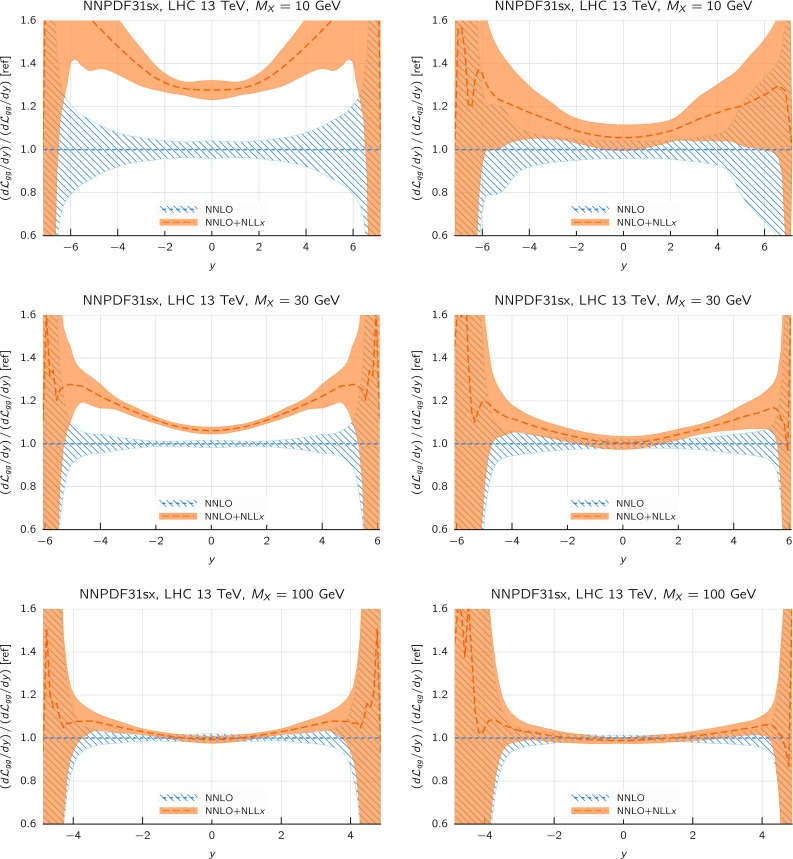
The double-differential PDF luminosities as a function of $\mu =M_X$ and *y*, Eq. (6.1), comparing the gluon–gluon (left plots) and quark–antiquark (right plots) luminosities between the NNLO and $\hbox {NNLO+NLL}x$ fits normalized to the central value of the former. We show the results as a function of *y* for $M_X=10,30,100$ GeV (top to bottom)

Now we move to compare PDF luminosities which are differential in rapidity, Eq. (6.1). As already mentioned, these luminosities allow for a more direct mapping between the final-state kinematics and the regions of $x,Q^2$ of the underlying PDFs. For simplicity, we focus here on the gluon–gluon and quark–antiquark luminosities, as the behavior of the gluon-quark and quark–quark is closely related to these two. In Fig. 31 we compare the PDF luminosities of the NNLO and $\hbox {NNLO+NLL}x$ fits, normalized to the central value of the former.

We show the results as a function of *y* for three different values of $M_X$, namely 10, 30, and 100 GeV.

From the comparisons of Fig. 31 we see that the impact of small-*x* resummation depends on the final-state rapidity *y*, and it increases close to the kinematic endpoints. This is expected as large (or small) values of the rapidity probe small-*x* values in one of the two partons that make up the parton luminosity, see Eq. (6.1). For instance, in the case of the *gg* luminosity, for the production of a final state with invariant mass $M_X=10 \, (30)$ GeV, the ratio between $\hbox {NNLO+NLL}x$ and NNLO is between 30 and 50% (10 and 20%), depending on the specific value of the rapidity. The differences are smaller in the case of the quark–antiquark luminosities, though we note that they could become more relevant if the PDF uncertainties were reduced by including in the fit data sensitive to the small-*x* region of the PDFs, such as the LHCb *W*, *Z* forward production cross-sections. This would, however, require the inclusion of small-*x* resummation in the partonic cross-sections for the relevant processes.

**Fig. 32:**
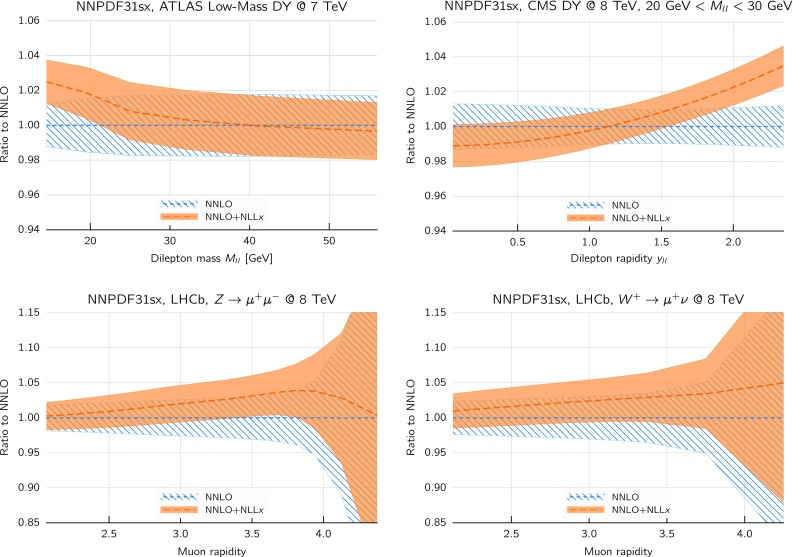
Comparison between the NNPDF3.1sx NNLO and $\hbox {NNLO+NLL}x$ predictions for selected Drell–Yan measurements at the LHC. From left to right and from top to bottom, we show the ATLAS low-mass measurements at 7 TeV, the CMS low-mass measurements at 8 TeV, and the LHCb $W^+$ and *Z* rapidity distributions at 8 TeV. For the $\hbox {NNLO+NLL}x$ predictions, the effects of small-*x* resummation are included in the PDF evolution but not in the partonic cross-sections

#### Implications for Drell–Yan production

We now present a first exploration of the possible phenomenological consequences of small-*x* resummation for LHC cross-sections, specifically for the Drell–Yan production process. We do this by providing estimates for some recent Drell–Yan cross-section measurements from the LHC, focusing on those more sensitive to the possible presence of small-*x* effects, and comparing the results of the predictions from the NNPDF3.1sx NNLO and $\hbox {NNLO+NLL}x$ fits, using in both cases the fixed-order NNLO hard-scattering cross-sections. These differences likely over-estimate the real effect, and in particular might be reduced once the resummation in the partonic cross-sections is taken into account [185]. However, we believe they provide a reliable though conservative estimate of the possible size of the resummation effects that can be expected.

Specifically, we show in Fig. 32 the predictions for the low-mass DY cross-sections from ATLAS at 7 TeV [156], the lowest invariant mass bin for the CMS Drell–Yan cross-sections double-differential in *y* and $M_{ll}$ at 8 TeV [195], as well as for forward $W^+$ and *Z* production at 8 TeV from LHCb [174]. Note that none of these datasets is included in the NNPDF3.1sx fits, since as discussed in Sect. 3 they are removed by the $H_{\mathrm{cut}}$ cut, Eq. (3.1). We stress once again that we calculate the $\hbox {NNLO+NLL}x$ and NNLO cross-sections using the corresponding PDFs from the NNPDF3.1sx fits, but using in both cases the fixed-order NNLO coefficient functions, using the same settings described in Sect. 3. We do not show the experimental data points in this comparison, as our aim is to focus on the impact of the resummation rather than a comparison with the measured cross-sections.

From the results shown in Fig. 32, we find that the NNLO and $\hbox {NNLO+NLL}x$ predictions are consistent within uncertainties in almost all cases. The differences are more marked for the kinematic regions directly sensitive to small-*x*, such as small $M_{ll}$ for the ATLAS data and large rapidities in the case of the LHCb and CMS measurements. In the latter case, the shift due to $\hbox {NNLO+NLL}x$ theory could be as large as $\sim 5\%$ at the largest rapidities, and the two PDF bands do not overlap for $y > 2$ in the invariant mass bin considered.

Moreover, since the experimental uncertainties for the cross-sections shown in Fig. 32 can be smaller than the corresponding PDF errors (and in some cases also smaller than the shift between the NNLO and $\hbox {NNLO+NLL}x$ curves), we can conclude from this exercise that the inclusion of these data into a fully consistent small-*x* resummed global PDF fit might provide further evidence for BFKL dynamics, this time from high-precision electroweak LHC cross-sections as opposed to from lepton–proton deep-inelastic scattering.

**Fig. 33:**
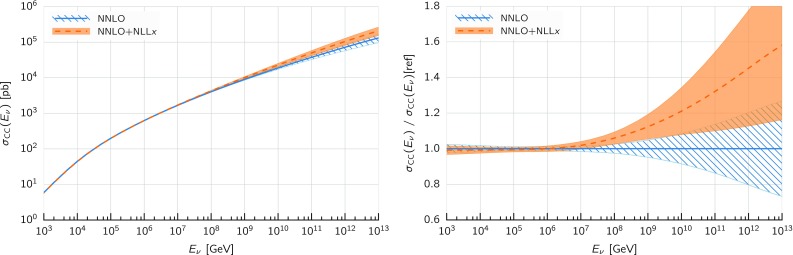
The UHE neutrino–nucleus charged-current cross-section $\sigma _{\mathrm{CC}}(E_{\nu })$ as a function of the neutrino energy $E_{\nu }$, comparing the results obtained using the NNPDF3.1sx NNLO fits with those of the its resummed $\hbox {NNLO+NLL}x$ counterpart

### The ultra-high-energy neutrino–nucleus cross-section

We next briefly explore the implication of the NNPDF3.1sx fits for the calculation of the total neutrino–nucleus cross-sections at ultra-high-energies (UHE). The interpretation of available and future UHE data from neutrino telescopes, such as IceCube [196] and KM3NET [197], requires precision predictions for the UHE cross-sections. With this motivation, a number of phenomenological studies of the UHE cross-sections and the associated uncertainties has been presented, both in the framework of collinear DGLAP factorization [198–204] and beyond it [205–209], the latter including for instance the effects of non-linear evolution or saturation.

Here we focus on the charged-current (CC) neutrino–nucleus inclusive cross-sections. Measuring neutrino–nucleus interactions at the highest values of $E_\nu$ accessible at neutrino telescopes explores values of *x* down to $\sim 10^{-9}$ for $Q\sim M_W$, thus representing a unique testing ground of small-*x* QCD dynamics. We have computed the theoretical predictions with APFEL+HELL for NNLO and $\hbox {NNLO+NLL}x$ theory, using the corresponding NNPDF3.1sx fits as input. Heavy-quark mass effects are included using the FONLL scheme, although these mass corrections are negligible at the relevant intermediate and high neutrino energies, so the calculation is effectively a massless one.

In Fig. 33 we show the UHE neutrino–nucleus charged-current cross-section $\sigma _{\mathrm{CC}}(E_{\nu })$ as a function of the neutrino energy $E_{\nu }$ for the fixed-order and for the resummed predictions. We show both the absolute cross-sections and the cross-sections normalized to the central value of the NNLO prediction. The error bands indicate the one-sigma PDF uncertainties. The upper limit in $E_{\nu }$ corresponds to the foreseeable range of the current generation of neutrino telescopes.

As we can see from the comparison of Fig. 33, the main effect of small-*x* resummation is to increase the cross-section at the highest energies, by an amount that can be as large as $50\%$ or more. The PDF errors are, however, large, and the NNLO and $\hbox {NNLO+NLL}x$ predictions agree at the one-sigma level on the whole range of energy considered. Given the large PDF uncertainties, it appears difficult to tell apart distinctive BFKL signatures in the total UHE inclusive cross-section. However, it is interesting to note that the effect of small-*x* resummation on the UHE cross-sections is the opposite of that obtained in calculations based on non-linear QCD dynamics, which instead predict a smaller cross-section at high energy (see e.g. [205]).

A promising strategy towards reducing the large PDF errors that affect $\sigma _{\mathrm{CC}}(E_{\nu })$ in Fig. 33 is provided by the inclusion of charm-production data from LHCb [210–212] in the PDF fits. As demonstrated in [14, 213, 214], the inclusion of LHCb *D*-meson production cross-sections gives a significant reduction in PDF uncertainties in the small-*x* region (up to an order of magnitude at $x\simeq 10^{-6}$), which in turns leads to UHE cross-sections with few-percent theory errors up to $E_{\nu }=10^{12}$ GeV [213]. In this respect, the combination of NLL*x* resummation and the additional constraints provided by the LHCb charm data would make possible a calculation of the UHE cross-sections with unprecedented theoretical and experimental uncertainties.

### Small-*x* resummation at future electron–hadron colliders

Since we have demonstrated the onset of BFKL dynamics in the inclusive HERA structure function data, it is natural to expect that the effects of small-*x* resummation will become even more relevant at the proposed future high-energy electron–hadron colliders: the higher their center-of-mass energy $\sqrt{s}$, the smaller the values of *x* kinematically accessible in the perturbative region of $Q^2$.

**Fig. 34:**
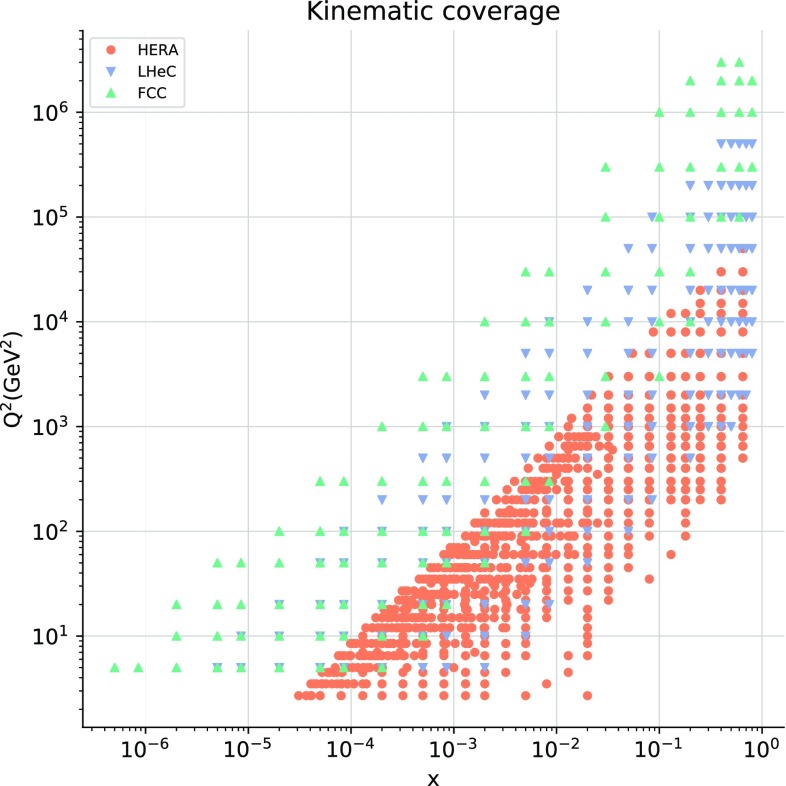
Kinematic coverage in the $(x,Q^2)$ plane of the FCC-eh and the LHeC experiments, compared to the kinematic coverage of the HERA structure function data

One such future *ep* collider is the large hadron-electron collider (LHeC) [191, 215]. In its latest design, a proton beam from the LHC with $E_{p}=7$ TeV would collide with an electron/positron beam with $E_e=60$ GeV coming from a new LinAc, thus enabling to access the region down to $x_{\mathrm{min}}\simeq 2\times 10^{-6}$ at $Q^2=2$ GeV$^2$. A more extreme incarnation of the same idea corresponds to colliding the same $E_e=60$ GeV electrons with the $E_p=50$ TeV beam of the proposed 100 TeV Future Circular Collider (FCC) [192, 193]. The resulting collider, dubbed FCC-eh, would be able to reach $x_{\mathrm{min}}\simeq 2\times 10^{-7}$ at $Q^2=2$ GeV$^2$. We show in Fig. 34 the kinematic coverage in the $(x,Q^2)$ plane of the two machines, compared with that of the HERA structure function data included in the NNPDF3.1sx fits. It is clear that these two machines would probe into the small-*x* region much deeper than HERA, thus allowing an unprecedented exploration of new QCD dynamics beyond fixed-order collinear DGLAP framework.

**Fig. 35:**
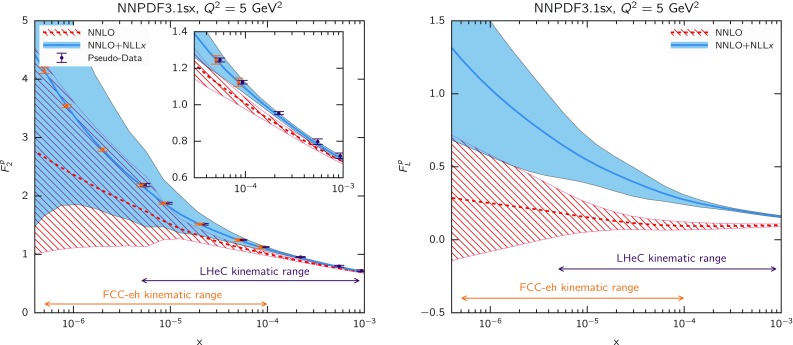
Predictions for the $F_2$ and $F_L$ structure functions using the NNPDF3.1sx NNLO and $\hbox {NNLO+NLL}x$ fits at $Q^2=5$ GeV$^2$ for the simulated kinematics of the LHeC and FCC-eh. In the case of $F_2$, we also show the expected total experimental uncertainties based on the simulated pseudo-data, assuming the $\hbox {NNLO+NLL}x$ values as central prediction. A small offset has been applied to the LHeC pseudo-data as some of the values of *x* overlap with the FCC-eh pseudo-data points. The inset in the left plot shows a magnified view in the kinematic region $x>3\times 10^{-5}$, corresponding to the reach of HERA data

In the following, we perform an initial exploration of the potential of the LHeC/FCC-eh for small-*x* studies. We use APFEL in conjunction with HELL to produce NNLO and $\hbox {NNLO+NLL}x$ predictions for various DIS structure functions, assuming the latest version of the simulated LHeC/FCC-eh kinematics.^7^ In Fig. 35 we show these predictions for the $F_2$ and $F_L$ structure functions using the NNPDF3.1sx NNLO and $\hbox {NNLO+NLL}x$ fits at $Q^2=5$ GeV$^2$ for the kinematics of the LHeC and the FCC-eh. In the case of $F_2$, we also show the expected total experimental uncertainties based on the simulated pseudo-data, assuming the $\hbox {NNLO+NLL}x$ curve as central prediction. To compare with the kinematic region within the reach of HERA data, we also show in the inset of the left plot the values of $F_2$ in the range $x> 3\times 10^{-5}$. The total uncertainties of the simulated pseudo-data are at the few-percent level at most; hence they are much smaller than the PDF uncertainties in most of the kinematic range. No simulated pseudo-data is currently available for $F_L$ using the latest scenarios for the two colliders; thus in this case we show only the theoretical predictions.

We now discuss in turn some of the interesting features in Fig. 35. First of all, we clearly see how with the FCC-eh one can probe the small-*x* region deeper than the LHeC by about an order of magnitude. Second, we find that the differences between NNLO and $\hbox {NNLO+NLL}x$ are moderate for $F_2$, especially if we take into account the large PDF uncertainties. The difference between the central values is in fact at the 15% level at $x\simeq 10^{-6}$, but the current PDF uncertainties are much larger. However, given the precision that the data could have, measuring $F_2$ (or alternatively the reduced cross-section $\sigma _{r, \mathrm NC}$) at the LHeC/FCC-eh would provide discrimination between the two theoretical scenarios of small-*x* dynamics. Indeed, we see that the differences between the central values of the fixed-order and resummed fits in the restricted kinematic region covered by HERA are already comparable or larger than the size of the simulated pseudo-data uncertainties.

This suggests that the inclusion of the LHeC/FCC-eh data for $F_2$ into a global fit would also provide discrimination power between the two theories, even if restricted to the HERA kinematic range. Finally, we see that differences are more marked for $F_L$, with central values differing by several sigma (in units of the PDF uncertainty) in a good part of the accessible kinematic range. This is yet another illustration of the crucial relevance of measurements of $F_L$ to probe QCD in the small-*x* region (as highlighted also by Fig. 21).

The comparisons of Fig. 35 do not do justice to the immense potential of future high-energy lepton–proton colliders to probe QCD in a new dynamical regime. A more detailed analysis, along the lines of Ref. [216], involves including various combinations of LHeC/FCC-eh pseudo-data ($\sigma ^\mathrm{red}_{\mathrm{NC}}, F_{L}, F_{2}^{c}$, etc.) into the PDF global analysis, allowing one to use the pseudo-data to reduce the PDF uncertainties and to quantify more precisely the discriminating power for small-*x* resummation effects with various statistical estimators, generalizing the analysis of the HERA data presented in Sect. 5. Such a program would illustrate the unique role of the LHeC/FCC-eh in the characterization of small-*x* QCD dynamics, and would provide an important input to strengthen the physics case of future high-energy lepton–proton colliders.

As a first step in this direction, we have performed variants of the NNPDF3.1sx fits including various combinations of the LHeC and FCC-eh pseudo-data of $\sigma ^\mathrm{red}_{\mathrm{NC}}$. Specifically, we have used the LHeC (FCC-eh) pseudo-data on $E_p=7$ (50) TeV + $E_e=60$ GeV collisions, where the central value of the pseudo-data has been assumed to correspond to the $\hbox {NNLO+NLL}x$ prediction computed with the corresponding resummed PDFs. All experimental uncertainties of the pseudo-data have been added in quadrature. The fits have been performed at the DIS-only level, since we have demonstrated in Sect. 5 that the small-*x* results are independent of the treatment of the hadronic data. Here we will show results of the fits including both LHeC and FCC-eh pseudo-data, other combinations lead to similar qualitative results.

**Table 7:** Same as Table 3 for the NNPDF3.1sx NNLO and $\hbox {NNLO+NLL}x$ fits including both the LHeC and the FCC-eh pseudo-data. We show only the $\chi ^2/N_{\mathrm{dat}}$ values for the HERA inclusive cross-sections and for the LHeC and FCC-eh pseudo-data, since for all other experiments the values presented in Table 3 are essentially unchanged. The last row corresponds to the sum of the three experiments listed on the table

	$N_{\mathrm{dat}}$	$\chi ^2/N_{\mathrm{dat}}$		$\Delta \chi ^2$
		NNLO	$\hbox {NNLO+NLL}x$	
HERA I+II incl. NC	922	1.22	1.07	$-138$
LHeC incl. NC	148	1.71	1.22	$-73$
FCC-eh incl. NC	98	2.72	1.34	$-135$
Total	1168	1.407	1.110	$-346$

First of all we discuss the fit results at the $\chi ^2/N_{\mathrm{dat}}$ level. For simplicity, we show only the results of the HERA inclusive cross-sections as well as that of the LHeC and FCC-eh pseudo-data: for all other experiments, the values presented in Table 3 are essentially unchanged. As shown in Table 7, it is not possible to find a satisfactory fit to the LHeC/FCC-eh pseudo-data on inclusive cross-sections using NNLO theory while assuming that $\hbox {NNLO+NLL}x$ theory is the correct underlying theory, as we have done here. As expected, the most marked differences are observed for the FCC-eh pseudo-data. Note that the last row in Table 7 corresponds to the sum of the three experiments listed on the table. By performing the same analysis as in Fig. 24, we have verified that the significant improvement in $\chi ^2/N_{\mathrm{dat}}$ between the NNLO and $\hbox {NNLO+NLL}x$ fits arises from the bins in the small-*x* and small-$Q^2$ region.

**Fig. 36:**
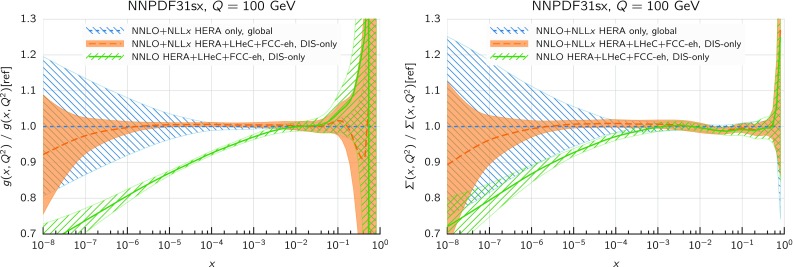
Comparison between the gluon (left plot) and the singlet (right plot) PDFs in the NNPDF3.1sx NNLO+NNL*x* fits without and with the LHeC+FCC-eh pseudo-data on inclusive structure functions. For completeness, we also show the results of the corresponding NNPDF3.1sx NNLO fit with LHeC+FCC-eh pseudo-data

Next in Fig. 36 we show the comparison between the gluon and the singlet PDFs at $Q=100$ GeV in the NNPDF3.1sx NNLO+NNL*x* fits without and with the LHeC+FCC-eh pseudo-data on inclusive structure functions. Note that the latter is a DIS-only fit, hence the differences observed at large-*x*. For completeness, we also show the results of the corresponding NNPDF3.1sx NNLO fit with LHeC+FCC-eh pseudo-data. In the case of the $\hbox {NNLO+NLL}x$, we see that the central values coincide within uncertainties (as expected by construction) and there is a significant uncertainty reduction both for the gluon and for the singlet. In particular, the LHeC+FCC-eh kinematic coverage ensures that a precision measurement of the small-*x* gluon, with few-percent errors down to $x\simeq 10^{-7}$, would be within reach.

From Fig. 36 we also see that for the gluon case, the NNLO and NNLO+NNL*x* fits with LHeC+FCC-eh pseudo-data are very different from each other. For instance, at $x\simeq 10^{-5}$, where we gluon can be pinned down with 1% errors, the central values of the two fits differ by $\sim 15\%$. This comparison highlights that the fixed-order description of the small-*x* region at these future high-energy colliders would be completely unreliable, and that accounting for the effects of resummation at small *x* is required for any quantitative prediction. Indeed, the LHeC and FCC-eh would be truly unique machines in their potential to unveil the new dynamical regimes of QCD that arise in the deep small-*x* region.

## Summary and outlook

The search for evidence of novel dynamics at small *x* beyond the linear fixed-order DGLAP framework has been an ongoing enterprise ever since the HERA collider started operations about 25 years ago. While some tantalizing hints have been reported, until now no conclusive evidence had been found in the HERA inclusive deep-inelastic structure functions. On the contrary, fixed-order perturbative QCD calculations have been remarkably successful, leading to good agreement with experimental data even in kinematic regions where they might naively be expected to fail.

From the theoretical point of view, formalisms for consistently including small-*x* resummation in DGLAP evolution and partonic coefficient functions were developed more than a decade ago [37–61]. While these were sufficient to explain the success of fixed-order perturbation theory in describing the data, a state-of-the-art global PDF fit including the effects of small-*x* resummation was never performed.^8^ It was the main goal of this study to bridge this gap, and to present the first genuine attempt at cutting-edge global NLO and NNLO PDF analyses which include the subtle effects of small-*x* resummation.

This has been made possible thanks to a number of developments both from the theory and from the implementation points of view. These include the consistent matching of NLL*x* small-*x* resummation to both the NLO and the NNLO fixed-order results, the resummation of the heavy-quark matching conditions and DIS coefficient functions, as well as the implementation of these theoretical developments in the public code HELL and its interface with the APFEL program [62, 63]. Also crucial to the success of the enterprise was the development of the NNPDF fitting technology, which is sensitive enough to identify small effects without them being masked by systematic methodological uncertainties from the fit procedure.

The main result of this work is the demonstration that including small-*x* resummation stabilizes the perturbative expansion of the DIS structure functions at small *x* and $Q^2$, and thus also of the PDFs extracted from them. Specifically, the PDFs obtained with small-*x* resummation using $\hbox {NLO+NLL}x$ and $\hbox {NNLO+NLL}x$ theory are in much closer agreement with each other at medium and small *x* than the corresponding fixed-order NLO and NNLO PDFs. This suggests in turn that the theoretical uncertainty due to missing higher order corrections in a $\hbox {NNLO+NLL}x$ resummed calculation is rather less at small *x* than that of the fixed-order NNLO calculation. This result is reflected in the marked improvement in the quantitative description of the HERA inclusive structure function data at small *x* when $\hbox {NNLO+NLL}x$ resummed theory is used rather than NNLO: the $\hbox {NNLO+NLL}x$ theory describes the low $Q^2$ and low *x* bins of the HERA data just as well as it describes the data at higher $Q^2$ and larger *x*. This effect is seen both in the inclusive neutral current and in the charm cross-sections, as expected from small-*x* resummation. We thus find no need for higher-twist contributions at low *x*, as proposed e.g. in Refs. [64, 69], at least in the region where the resummed perturbative calculation is valid.

We have also presented here a first exploration of the phenomenological implications of our results. It has been understood for some time that the effect of resummation on the evolution of the PDFs can have a significant impact on the shape of parton luminosities and thus of hadronic cross-sections at LHC [44]. We have now shown that the further effect on parton luminosities of including small-*x* resummation in a PDF fit to low-$Q^2$ data at small *x* also remains sizable even at higher scales. We therefore expect that at the LHC small-*x* resummation might have significant effects, at either low invariant masses or at high rapidities, and thus that the accurate description of processes in these kinematic regions will require small-*x* resummation Conversely, present and future LHC measurements might provide further evidence for the onset of BFKL dynamics, this time in proton-proton collisions.

Small-*x* resummation also plays a crucial role in shaping the physics case for future high-energy lepton–proton colliders such as the LHeC and the FCC-eh, which would extend the coverage of HERA by up to two orders of magnitude into the small-*x* region. In this respect, the NNPDF3.1sx fits can be used to improve the accuracy of existing calculations of deep-inelastic scattering processes at these new machines. We have also demonstrated that a clear probe of BFKL dynamics is provided by the UHE neutrino–nucleus cross-sections, where differences in event rates could be observed by upcoming measurements with neutrino telescopes such as IceCube and KM3NET.

The main limitation of the present analysis is the need to impose stringent cuts to the fitted hadronic data, in particular for Drell–Yan production, in order to ensure that the contamination from unresummed partonic cross-sections is kept to a minimum. On the one hand, it is well understood how to combine resummation corrections to partonic cross-sections with resummed parton luminosities to obtain fully resummed cross-sections even when the coupling runs [44], and small-*x* resummed partonic cross-sections have been computed for many of the relevant collider processes  [100, 101, 104–108, 111–117]. On the other hand, these calculations are still not available in a format amenable to systematic phenomenology, and some effort is still required before they can be used in PDF fits. Future work in this direction will allow us to include a wider range of hadron collider data into a fully consistent small-*x* resummed global fit by removing the need for such cuts, and therefore allow us to achieve the same experimental precision for the resummed PDFs as is now possible in fixed-order fits. Moreover, an accurate description of processes at high rapidity, such as forward Drell–Yan and *D* meson production at LHCb, is likely to require the simultaneous resummation of both small-*x* and large-*x* logarithms, since at high rapidity while one of the partons is at very small *x*, the other is at very large *x*.

Finally, we would like to emphasize that the implications of our results go beyond what is traditionally thought of as “small-*x* physics”. As LHC data become ever more precise, the theoretical challenge is to reduce theoretical uncertainties down to the 1% level, and this will require consistent calculations in perturbative QCD at $\hbox {N}^3\hbox {LO}$. Recent progress with four-loop splitting functions [86, 87] suggests that this may be possible rather sooner than was previously thought. However, while at NNLO the most singular term in the gluon splitting function is of order ${{\alpha _s^3}\over {x}}\ln {{1}\over {x}}$ (the term with two logarithms being accidentally zero), at $\hbox {N}^3\hbox {LO}$ the most singular term is or order ${{\alpha _s^4}\over {x}}\ln ^3{{1}\over {x}}$. We thus expect the instability in fixed-order perturbative evolution at small *x* to be rather worse at $\hbox {N}^3\hbox {LO}$ than it was at NNLO. Small-*x* resummation would then be mandatory for improved precision, and this would require $\hbox {N}^3\hbox {LO}+\hbox {NNLL}x$ calculations to properly resum all the small *x* logarithms. While there has been some progress in extending the BFKL kernel to $\hbox {NNLL}x$ [88–94], much work remains to be done.

*Delivery*

The fits presented in this work are available in the LHAPDF6 format [217] from the webpage of the NNPDF collaboration: http://nnpdf.mi.infn.it/nnpdf3-1sx These sets are based on the global dataset and contain $N_{\mathrm{rep}}=100$ replicas. Specifically, the following fits are available:

- Baseline NLO and NNLO NNPDF3.1sx sets, which are based on the global dataset with the kinematical cut of $H_{\mathrm{cut}}=0.6$ applied to the hadronic data: NNPDF31sx_nlo_as_0118
NNPDF31sx_nnlo_as_0118
- Resummed $\hbox {NLO+NLL}x$ and $\hbox {NNLO+NLL}x$ NNPDF3.1sx sets, which are the resummed counterparts of the baseline sets above, based on an identical input dataset with the only difference of the theory settings:
NNPDF31sx_nlonllx_as_0118
NNPDF31sx_nnlonllx_as_0118

In addition, the other NNPDF3.1sx fits presented in this work, such as the DIS-only fits, are available upon request from the authors.

The DIS-only fits with various combinations of the LHeC and FCC-eh pseudo-data, discussed in Sect. 6.3, are also available in the same webpage:

NNPDF31sx_nnlo_as_0118_DISonly_LHeC

NNPDF31sx_nnlo_as_0118_DISonly_FCC

NNPDF31sx_nnlo_as_0118_DISonly_FCC+LHeC

NNPDF31sx_nnlonllx_as_0118_DISonly_LHeC

NNPDF31sx_nnlonllx_as_0118_DISonly_FCC

NNPDF31sx_nnlonllx_as_0118_DISonly_FCC+LHeC

## Footnotes

1 It has been shown that the description of these data improves if either a higher-twist term or a phenomenological higher-order correction to $F_L$ is included [64].

2 In its current implementation, HELL 2.0 can only be used for values of *Q* such that $\alpha _s(Q)\le 0.35$.

3 Note that when the charm PDF is fitted, this manipulation is not really legitimate, as contributions from an “intrinsic” component would be suppressed by the damping but may not be subleading.

4 As observed in Ref. [79], the fit quality to the charm data in NNLO global fits improves if the charm is perturbatively generated, but it leads to a significant overall deterioration of the global $\chi ^2$ with respect to a fit where the charm is independently parametrized.

5 The $F_L$ structure function has also been measured by the ZEUS collaboration [189], but with a reduced kinematic coverage of the small-*x* region. The ZEUS measurement is in mild tension with the H1 measurement, though it is affected by larger experimental uncertainties.

6 The H1 measurement includes three further bins at small $Q^2$, reaching down to $Q^2=1.5$ GeV$^2$.

7 We thank Max Klein for providing us with the LHeC/FCC-eh pseudo-data.

8 A global fit including the effect of small-*x* resummation was performed in Ref. [61] more than a decade ago. Although the framework for implementing small-*x* resummation was not the same that we use, and the dataset could not contain LHC data at that time, the results were similar; in particular including the resummation improves the fit quality.
